# Supplementary material for: The genomic landscape of molecular responses to natural drought stress in Panicum hallii
Source: Nat Commun. 2018 Dec 6;9:5213. doi: 10.1038/s41467-018-07669-x (PMC6283873; doi:10.1038/s41467-018-07669-x)
Supplement: Supplementary file 1 — Supplementary Information [file 41467_2018_7669_MOESM1_ESM.docx]

**The genomic landscape of molecular responses to natural drought stress in *Panicum hallii***

Lovell *et al.*

**Supplementary Note 1. Genome assembly details**

**Sequencing of FIL2**

We sequenced *Panicum hallii var. filipes* (genotype *FIL2*) using a whole genome shotgun sequencing strategy and standard sequencing protocols. Sequencing reads were collected using Illumina, PACBIO, Moleculo, and Sanger platforms. Illumina, PACBIO, and Sanger reads were sequenced at the Department of Energy (DOE) Joint Genome Institute (JGI) in Walnut Creek, California and the HudsonAlpha Institute in Huntsville, Alabama. Sanger BACs were sequenced using an ABI 3730XL capillary sequencer. Illumina reads were sequenced using the Illumina HISeq platform, and the PACBIO reads were sequenced using the RSII platform. One 250bp insert 2x150 Illumina fragment library (92.1x) was sequenced. Prior to assembly, Illumina reads were screened for mitochondria, chloroplast, and other contamination. Reads composed of >95% simple sequence were removed. Illumina reads <50bp after trimming for adapter and quality (q<20) were removed. The final read set consists of 328,922,392 reads for a total of 92.10x of high quality Illumina bases. For the PACBIO sequencing, a total of 36 P5C2 chips and 41 P6C4 chips (4 hour movie time) were sequenced with a p-read yield of 47.93 Gb, with a total coverage of 95.87x.

**Genome assembly and construction of pseudomolecule chromosomes for FIL2**

An improved version 3.0 assembly was generated by assembling the 4,520,785 PACBIO reads (95.87x sequence coverage) using the MECAT assembler^1^ and subsequently polished using QUIVER^2^. This produced 1,664 scaffolds (1,664 contigs), with a contig N50 of 654.5 Kb, 1,059 scaffolds larger than 100 Kb, and a total genome size of 539.9 Mb.

A 325,613 marker map provided by Tom Juenger (UT Austin) was aligned to the *FIL2* MECAT assembly to form chromosomes. Misjoins were characterized as a discontinuity in the *FIL2* linkage group. A total of 115 breaks were identified and made. The *FIL2* scaffolds were then oriented, ordered, and joined together into 9 chromosomes using the map. A total of 1,246 joins were made during this process. Each chromosome join is padded with 10,000 Ns. Significant telomeric sequence was identified using the TTTAGGG repeat, and care was taken to make sure that it was properly oriented in the production assembly. The remaining scaffolds were screened against bacterial proteins, organelle sequences, GenBank nr and removed if found to be a contaminant. After forming the chromosomes, it was observed that some small (<20Kb) redundant sequences were present on adjacent contig ends within chromosomes. To fix this problem, adjacent contig ends were aligned to one another using BLAT^3^, and duplicate sequences were collapsed to close the gap between them. A total of 450 adjacent contig pairs were collapsed.

Post collapsing adjacent contigs, a set of 30,315 (430.3 Mb) clone sequences, along with a set of 704,618 (3.74 Gb, 6.9x coverage) Moleculo reads were used to patch gaps. Gaps were patched by first breaking the chromosomes into contigs. 1.5 kb of sequence was trimmed off of the contig ends and the trimmed portion was broken into 100mers. The 100mers were aligned to the clones/moleculo using blastn, and clones/moleculo reads were associated with specific gaps. Clones/moleculo reads spanning a gap were then used to patch the gap. A total of 60 of the remaining gaps were patched. Finally, homozygous SNPs and INDELs were corrected in the release consensus sequence using ~50x of Illumina reads (2x150, 400bp insert) by aligning the reads using bwa mem^4^ and identifying homozygous SNPs and INDELs with the GATK’s UnifiedGenotyper tool^5^. A total of 68 homozygous SNPs and 10,935 homozygous INDELs were corrected in the release. The final version 3.0 release contains 528.5 Mb of sequence, consisting of 1,027 contigs with a contig N50 of 1.1 Mb and a total of 94.8% of assembled bases in chromosomes.

Completeness of the euchromatic portion of the version 3.0 assembly was assessed using 33,004 primary transcripts >500bp from the version 2.0 FIL2 release. The aim of this analysis is to obtain a measure of completeness of the assembly, rather than a comprehensive examination of gene space. The transcripts were aligned to the assembly using BLAT^3^ and alignments >=95% base pair identity and >=95% coverage were retained. The screened alignments indicate that 32,976 (99.92%) of the version 2.0 FIL2 primary transcripts aligned to the version 3.0 release. Of the unaligned 28 transcripts, 27 of them were not anchored in a chromosome in the version 2.0 release.

**Construction of the scaffold assembly for FIL2**

A total of 4,520,785 PACBIO reads (95.87x) were assembled using MECAT^1^, and formed the starting point of the version 3.0 release. The 328,922,392 Illumina sequence reads (92.1x sequence coverage) was used for fixing homozygous snp/indel errors in the consensus. Gaps were patched in the final version 3.0 release using the 6.9x Moleculo read set.

**Screening and final assembly release of FIL2**

Scaffolds that were not anchored in a chromosome were classified into bins depending on sequence content. Contamination was identified using blastn against the NCBI nucleotide collection (NR/NT) and blastx using a set of known microbial proteins. Additional scaffolds were classified as repetitive (>95% masked with 24mers that occur more than 4 times in the genome) (149 scaffolds, 4.3 Mb), alternative haplotypes (unanchored sequence with >95% identity and >95% coverage within a chromosome) (41 scaffolds, 1.5 Mb), chloroplast (13 scaffolds, 718.3 Kb), mitochondria (10 scaffolds, 672.2 Kb), and low quality (>50% unpolished bases post polishing, 9 scaffolds, 114.9 Kb).

**Assessment of assembly accuracy of FIL2**

A set of 543 contiguous BAC clones >100Kb were selected in order to assess the accuracy of the assembly. A range of variants were detected in the comparison of the BAC clones and the assembly. In 507 of the BAC clones, the alignments were of high quality (< 0.05% bp error) with an example being given in Supplementary Fig. 3. The remaining 36 BACs indicate a higher error rate due mainly to their placement in more repetitive regions. The overall bp error rate in the BAC clones is 0.062% (43,302 discrepant bp out of 69,469,413).

**Sequencing of HAL2**

We sequenced *Panicum hallii var. hallii* (genotype *HAL2*) using a whole genome shotgun sequencing strategy and standard sequencing protocols. Sequencing reads were collected using Illumina and PACBIO. Illumina and PACBIO were sequenced at the Department of Energy (DOE) Joint Genome Institute (JGI) in Walnut Creek, California and the HudsonAlpha Institute in Huntsville, Alabama. Illumina reads were sequenced using the Illumina HISeq platform, and the PACBIO reads were sequenced using the RSII platform. One 800bp insert 2x250 Illumina fragment library (152.3x) was sequenced. Prior to assembly, Illumina reads were screened for mitochondria, chloroplast, and other contamination. Reads composed of >95% simple sequence were removed. Illumina reads <75bp after trimming for adapter and quality (q<20) were removed. The final read set consists of 296,294,162 reads for a total of 152.3x of high quality Illumina bases. For the PACBIO sequencing, a total of 56 P6C4 chips (10 hour movie time) were sequenced with a p-read yield of 44.76 Gb, with a total coverage of 89.53x.

**Genome assembly and construction of pseudomolecule chromosomes for HAL2**

An improved version 2.0 assembly was generated by assembling the 3,280,170 PACBIO reads (89.53x sequence coverage) using the MECAT assembler^1^ and subsequently polished using QUIVER^2^. This produced 190 scaffolds (190 contigs), with a contig N50 of 7.2 Mb, and a total genome size of 488.2 Mb. A 325,613 marker map provided by Tom Juenger (UT Austin) was aligned to the HAL2 MECAT assembly to form chromosomes. Misjoins were characterized as a discontinuity in the HAL2 linkage group. A total of 1 break was identified and made. The HAL2 scaffolds were then oriented, ordered, and joined together into 9 chromosomes using the map. A total of 119 joins were made during this process. Each chromosome join is padded with 10,000 Ns. Significant telomeric sequence was identified using the TTTAGGG repeat, and care was taken to make sure that it was properly oriented in the production assembly. The remaining scaffolds were screened against bacterial proteins, organelle sequences, GenBank nr and removed if found to be a contaminant. After forming the chromosomes, it was observed that some small (<20Kb) redundant sequences were present on adjacent contig ends within chromosomes. To fix this problem, adjacent contig ends were aligned to one another using BLAT^3^, and duplicate sequences were collapsed to close the gap between them. A total of 18 adjacent contig pairs were collapsed. Finally, homozygous SNPs and INDELs were corrected in the release consensus sequence using ~50x of Illumina reads (2x250, 800bp insert) by aligning the reads using bwa mem^4^ and identifying homozygous SNPs and INDELs with the GATK’s UnifiedGenotyper tool^5^. A total of 188 homozygous SNPs and 16,991 homozygous INDELs were corrected in the release. The final version 2.0 release contains 486.5 Mb of sequence, consisting of 144 contigs with a contig N50 of 8.3 Mb and a total of 99.39% of assembled bases in chromosomes.

Completeness of the euchromatic portion of the version 2.0 assembly was assessed using 33,004 primary transcripts >500bp from the version 2.0 FIL2 release. The aim of this analysis is to obtain a measure of completeness of the assembly, rather than a comprehensive examination of gene space. The transcripts were aligned to the assembly using BLAT and alignments >=95% base pair identity and >=95% coverage were retained. The screened alignments indicate that 32,816 (99.40%) of the version 2.0 FIL2 primary transcripts aligned to the version 2.0 HAL2 release. Of the unaligned 188 transcripts, 32 of them were not anchored in a chromosome in the version 2.0 FIL2 release.

**Construction of the scaffold assembly for HAL2**

A total of 3,280,170 PACBIO reads (89.53x) were assembled using MECAT, and formed the starting point of the version 2.0 release. The 296,294,162 Illumina sequence reads (152.3x sequence coverage) was used for fixing homozygous snp/indel errors in the consensus.

**Screening and final assembly release for HAL2**

Scaffolds that were not anchored in a chromosome were classified into bins depending on sequence content. Contamination was identified using blastn against the NCBI nucleotide collection (NR/NT) and blastx using a set of known microbial proteins. Additional scaffolds were classified as repetitive (>95% masked with 24mers that occur more than 4 times in the genome) (24 scaffolds, 1.2 Mb), unanchored rDNA (1 scaffold, 51.5 Kb), low quality (>50% unpolished bases post polishing, 1 scaffolds, 33.0 Kb), chloroplast (3 scaffolds, 174.0 Kb).

**Supplementary Note 2. Genome annotation details**

**Overview**

The ability to produce comprehensive RNA-seq data at low cost has greatly benefitted gene structure prediction, facilitating the prediction of UTR and alternative splice isoforms, compared to cases where prediction was dominated by protein homology and relatively sparse EST sets. The accurate and time-efficient construction of RNAseq based transcript assemblies is an absolute requirement for taking advantage of RNA-seq data in gene prediction, and is an area of active research, with several programs already available.

PERTRAN is a pipeline for assembling transcripts from RNA-seq reads which demonstrates high sensitivity, with few fused exons fast run times and is implemented in PERL following three steps: 1) transcript assembler (GSNAP SAM parser) 2) re-assembly, and 3) read alignment via GSNAP (Wu, 2010). Parallelization was achieved by splitting a FASTAQ file into multiple GSNAP jobs while trimming off very low-quality bases and assembling transcripts in many overlapping segments per chromosome.

**Pipeline**

We first align reads to reference genome by GSNAP and parse mapping data to the genomic position (with pairing position and/or with splice position(s), if any), in GFF2 format. The number of mapped reads is recorded as the expression level for each genomic position. Gap position and number of pairs are recorded if the distance between paired reads is larger than a threshold (gap length threshold), normally read pair inner distance average plus 1 variance, and so are introns from splice-aware-aligned reads (spliced reads). We then produce raw read contigs, by combining paired reads that map within a threshold gap distance, which accounts for splice sites. Any continuous block with expression across a chromosome is a raw read contig. The raw contig is un-stranded unless reads are sequenced with a stranded protocol.

We check if contigs could be split into multiple contigs if expression level in a gap(s) or intron(s) suggests intron(s). Each position expression of a raw contig is deducted from the average expression in gap(s) or intron(s) and a continuous block of expression level below zero is a putative intron. Splice sites between any 2 contigs of inner distance ≤ maximum intron length (a user parameter) with the number of gaps/introns higher than a threshold (default: 3 gaps or 1 spliced read for seeding) are computed using position weight matrix (PWM) either from priori spliced site sequences or canonical splice empirical distribution (donor: 0.993 GT, 0.006 GC, 0.001 AT, acceptor: 0.999 AG, 0.001 AC). Splice site score is computed from PWM and the score is penalized by its position relative to contig edge with +9 from left side (or -9 from right side) as center with no penalty. Splice site score based on PWM is useful when implying splice sites using short read pairing information. Such splice inference is turned off as default currently because long reads are readily available. Score is set to an arbitrary high number if splice site is supported by spliced reads.

An intron is made from two sensical splice sites with the highest score total for any given two contig pair and additional intron(s) if supported by spliced reads. An intron can also be made from other rare splice sites (not GT/AG, GC/AG, AT/AC) if supported by spliced reads and intron score is more than 0.65. The intron score is computed as follows: Consensus sequences of splice 5’ site, 3’ site and branch point for U2 type and U12 type spliceosomes are used to fill scoring matrices. Intron score is the number of hydrogen bonds instead of just simple base matches (match of G or C counting for 3 while A or T for 2) and normalized to the total number of possible hydrogen bonds (if all bases had been matched). For >1 introns per contig pair to be retained, all introns must be supported by spliced reads. Transcript graphs with exons as nodes and introns as edges are made.

Each transcript graph with alternative path as alternative transcript is trimmed if not meeting minimum of minor transcript ratio to major (default 10%) in the number of spliced reads, in the number of gaps with minimum of 0.1% of overall spliced reads per site in the transcript or minimum spliced read threshold, or in overall expression level with spliced reads with minimum spliced read threshold. Overlapping introns on opposite strands of lower number of spliced reads or gaps are removed unless the number of spliced reads exceeds a threshold (default is number of spliced reads for seeding plus 2). Two terminal overlapping exons on opposite strand are shrunk if expression level drops by >2X in the middle so 2 exons would not overlap using the lowest level as the split point.

For computation speed, overlapping chromosome segments (default 10M BP with 1K BP overlapping) are used in transcript computation resulting in a few overlapping partial transcripts. Overlapping transcripts on the same strand are merged if their ends are different and their overlapping exons match. Terminal exons are extended if supported by expression using raw contigs and the extension wouldn’t run into another assembly. Furthermore, terminal exons are shrunk from either end until base expression level exceeds exon average expression level times half of minor transcript ratio (default is 5% of average expression level).

**Supplementary Note 3. Comparative genomics pipeline**

Here we present the stepwise GENESPACE pipeline.

First, to develop high-confidence, non-overlapping collinear blocks, we extract primary transcript peptide sequence for each gene model in each annotation, then conduct reciprocal pairwise (and intra-genomic) BLASTp searches between all genome annotations. BLASTp is implemented via diamond within orthofinder

We then cull BLAST results to high-quality hits, above an absolute score (default = 100), and score relative to the best hit for the gene (default = 50%). Hits also must correspond with groupings in the orthofinder orthogroup output. Finally, we check for density of hits in x-y space (n = hits with r = rank distance radius), where each axis is the positional rank of each gene in the BLAST hit (default = [n = 10,10,10],[r = 100,50,20]) using DBScan.

We find collinear blocks of genes from culled BLASTp hits, via MCScanX using the following parameters: -s 5 (min block size) -m 10 (maximum number of gaps) -a (only ouput collinearity file). We then merge overlapping MCScanX blocks, so that no block exists entirely (or partially) within the xy gene-position rank space of another block; blocks overlapping by > 1 ordered hit are merged. Finally, we parse the block breakpoints so that the minimal total number of breakpoints are required to describe all blocks across all genomes.

To find orthologous sequences within collinear blocks, we extract assembly genomic DNA sequence for all genomes within each breakpoint and repeat orthofinder run as above. We then parse orthogroups, splitting into three groups: 1) Private orthogroups – these are single or clusters of genes that never have representation of any other genomic blocks. The ancestral state of these genes cannot be inferred. These genes are stored in a text file and not used again. 2) Complete orthogroups. These have at least one gene model from each genomic block. These are aligned and written to file. 3) Incomplete orthogroups, which have representation of >1 genomic block, but not all of the blocks. For each incomplete orthogroup, blat exons from mapped genes against unmapped assembly block. Parse blat output to find the most likely location of a gene, allowing up to 50kb introns. Finally, extract the genomic sequence of the likely location of the gene sequence and run exonerate against the cds using the following parameters: --model est2genome --forcescan target –softmasktarget --gappedextension --refine region --dpmemory 2000 --maxintron 10000 -n 1--showalignment no --showtargetgff yes --showvulgar no --showcigar no --ryo ">%qi\n%tas\n" --alignmentwidth 1000000

We then include the exonerate ouptut in the orthogroup fasta and generate multiple sequence alignment of all orthogroup sequences via MAFFT.

**Supplementary Figure 1.** Divergence time estimates for each of the six subpopulations of *P. hallii*. Solid, opaque lines are the means of 100 bootstraps. Transparent grey lines are the individual bootstrap estimates of effective population size over time. The time (x) axis is log transformed. Panels are colored by the variety, where FIL = *var. filipes* and HAL = *var. hallii*. The y-axis (proportion of relative cross coalescence is thresholded at 0.25 so all panels can be interpreted together. Without thresholding, closely related subpopulations (e.g. bottom right) reach 1 quickly. Data presented herein can be found in Supplementary Data 2.

**Supplementary Figure 2.** Effective population size estimates for each of the six subpopulations of *P. hallii*. Solid, opaque lines are the means of 100 bootstraps. Transparent grey lines are the individual bootstrap estimates of effective population size over time. Both the x- and y-axes are log transformed. Panels are colored by the variety, where FIL = *var. filipes* and HAL = *var. hallii*. Data presented herein can be found in Supplementary Data 2.

| 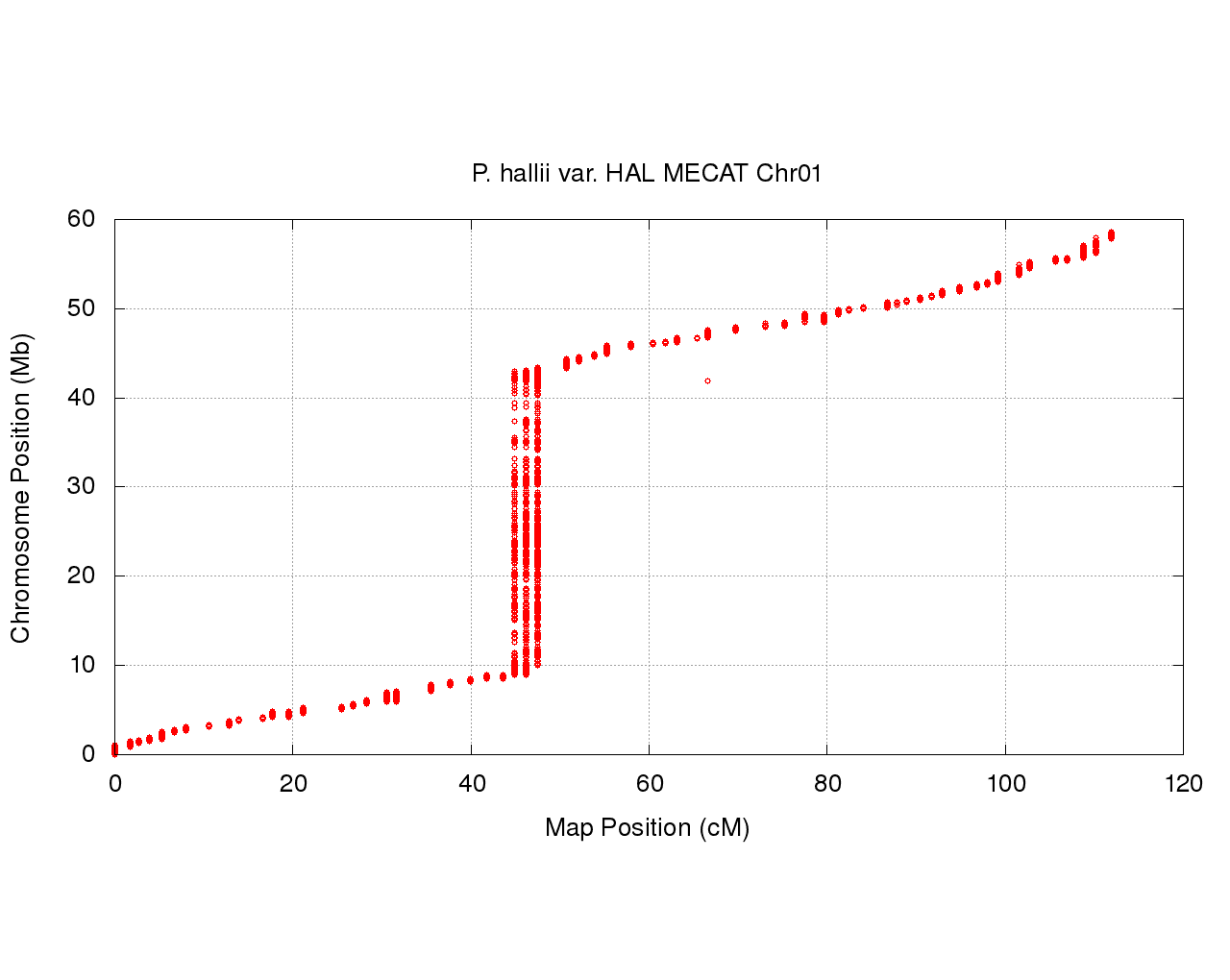 | 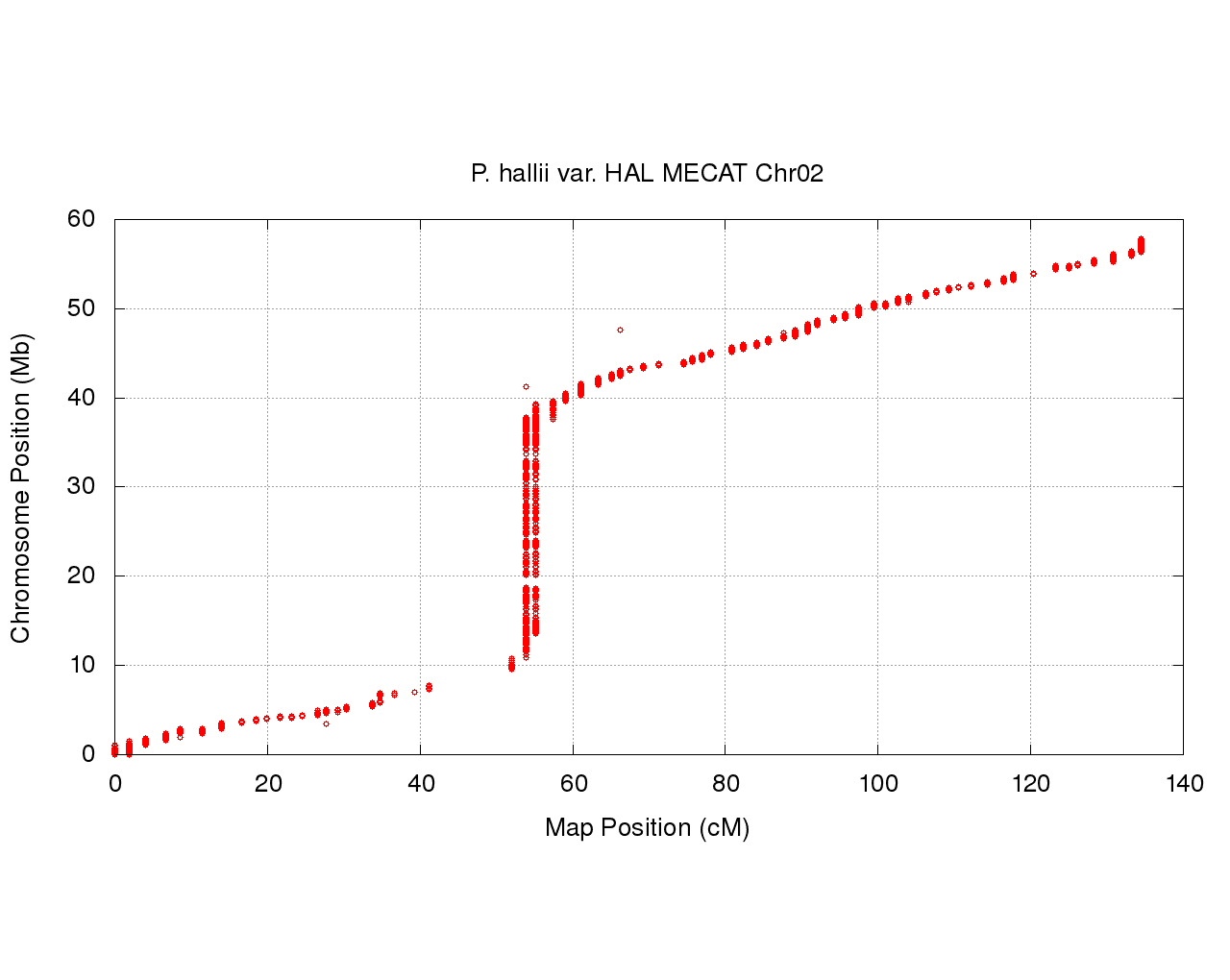 | 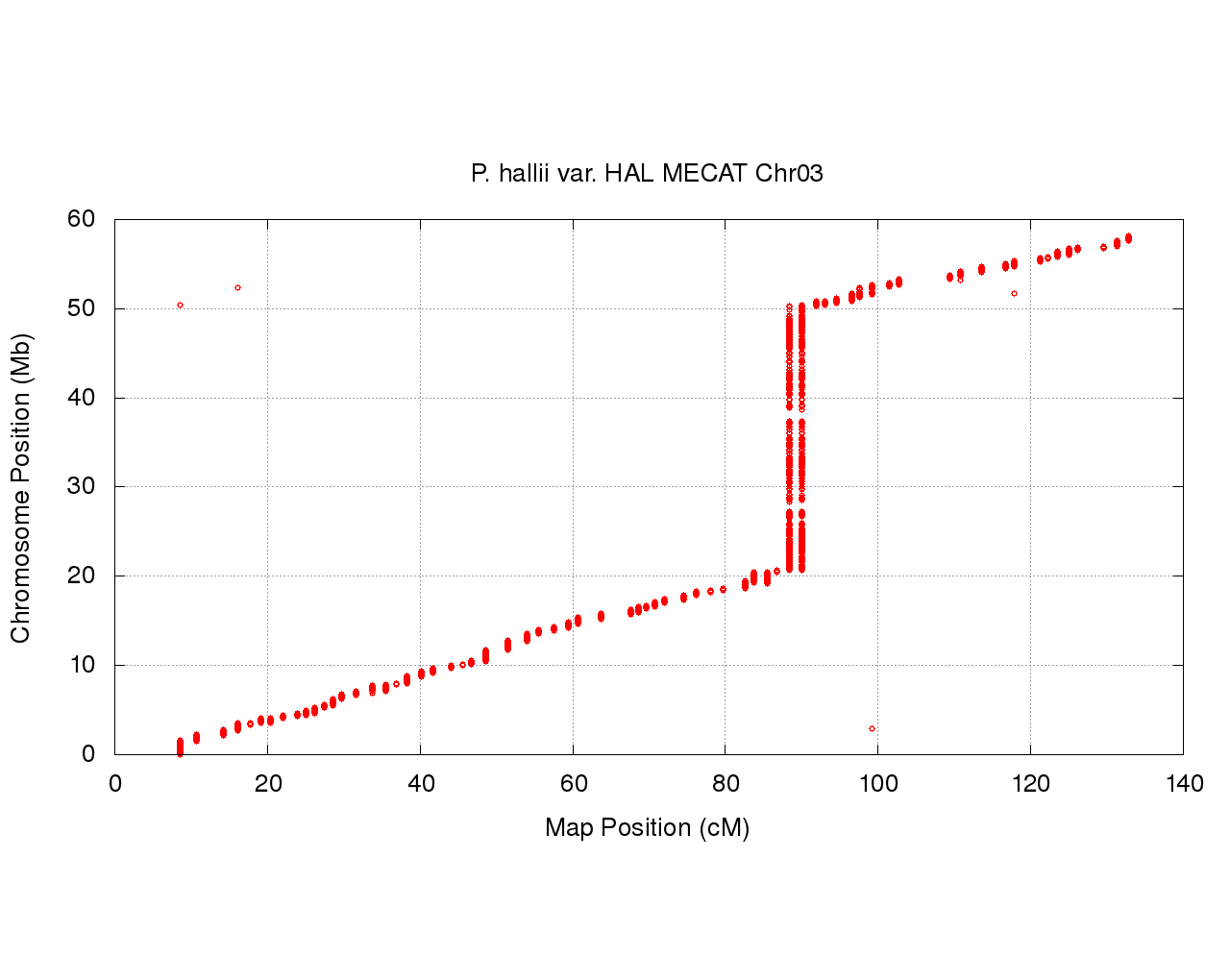 |
| --- | --- | --- |
| 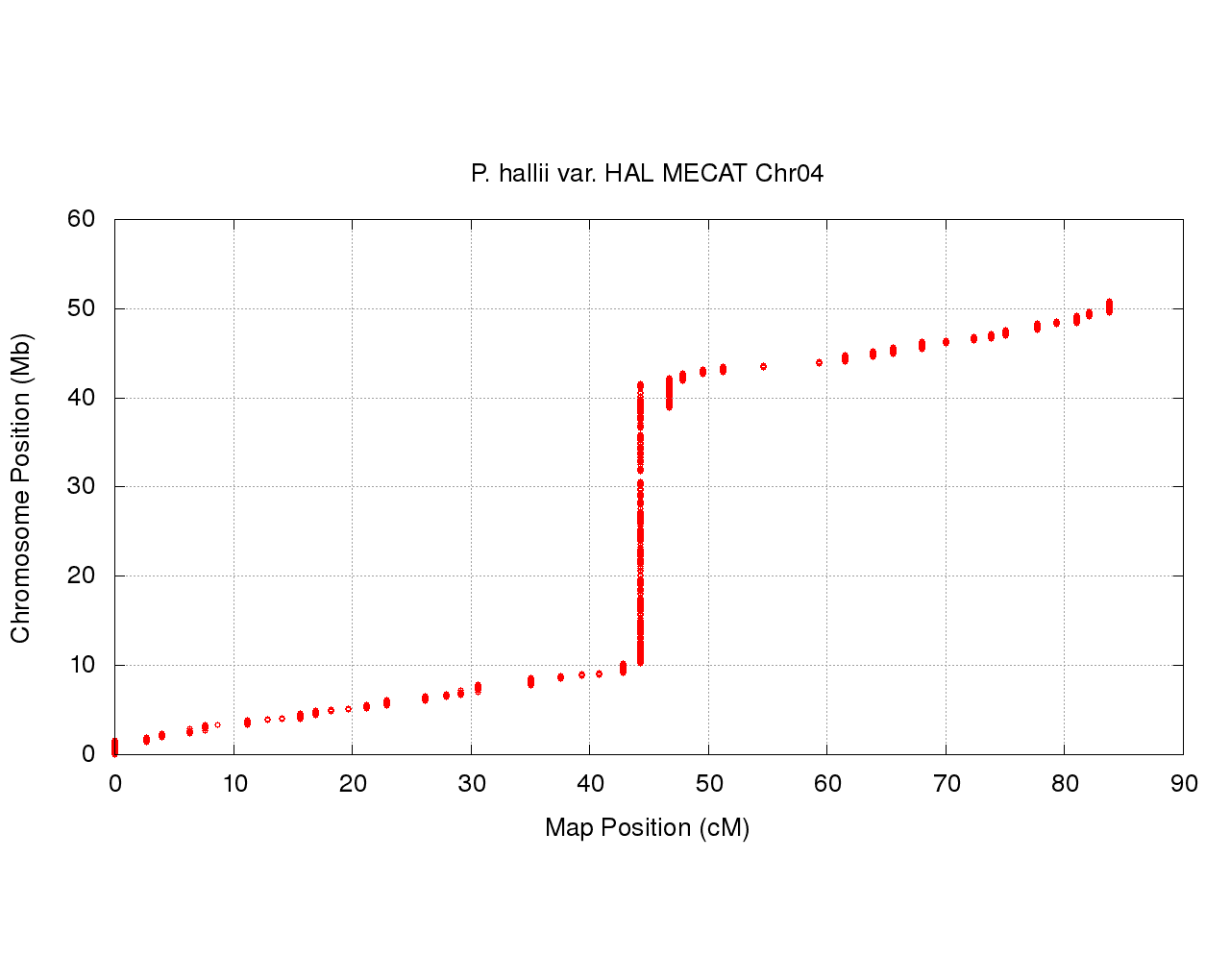 | 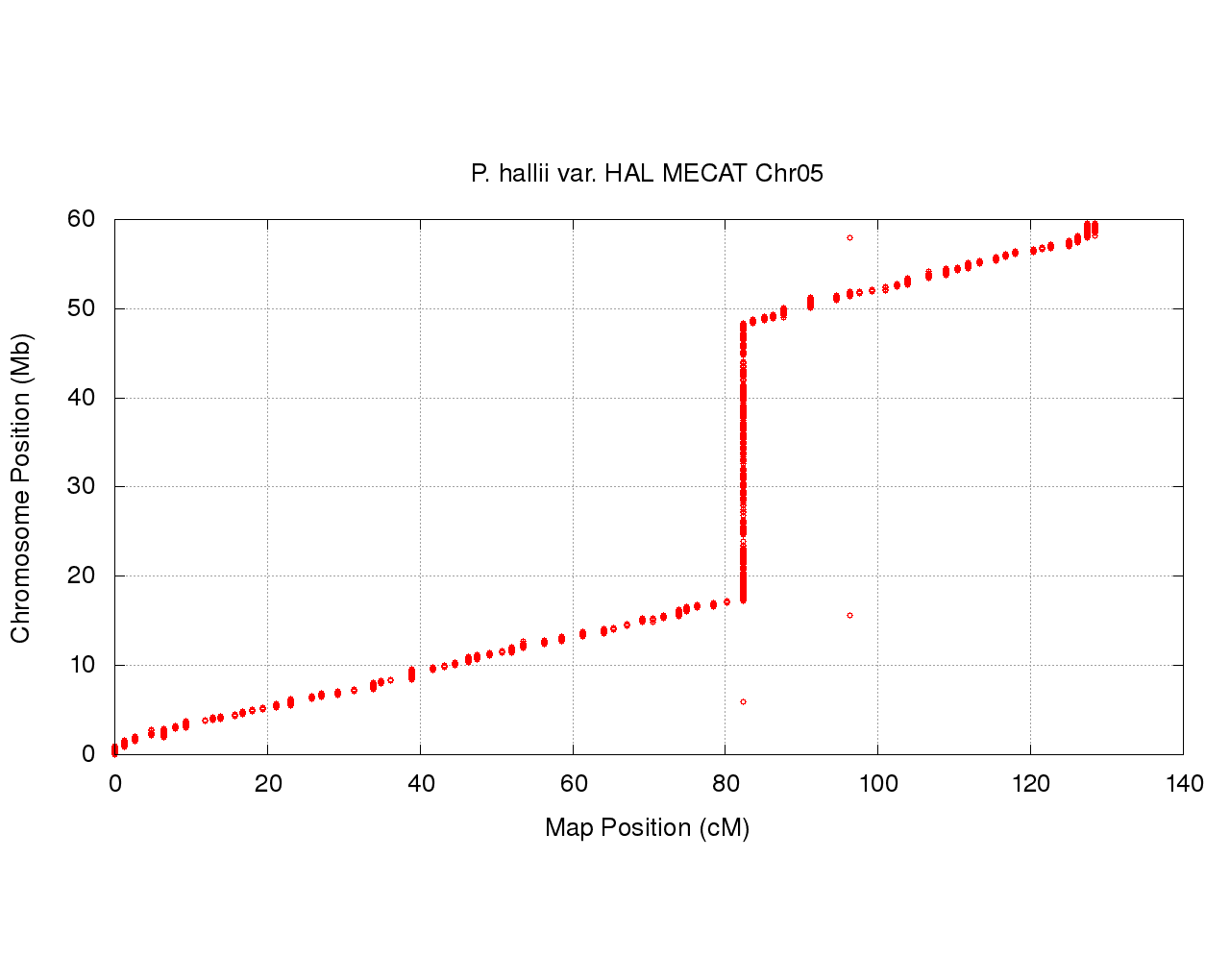 | 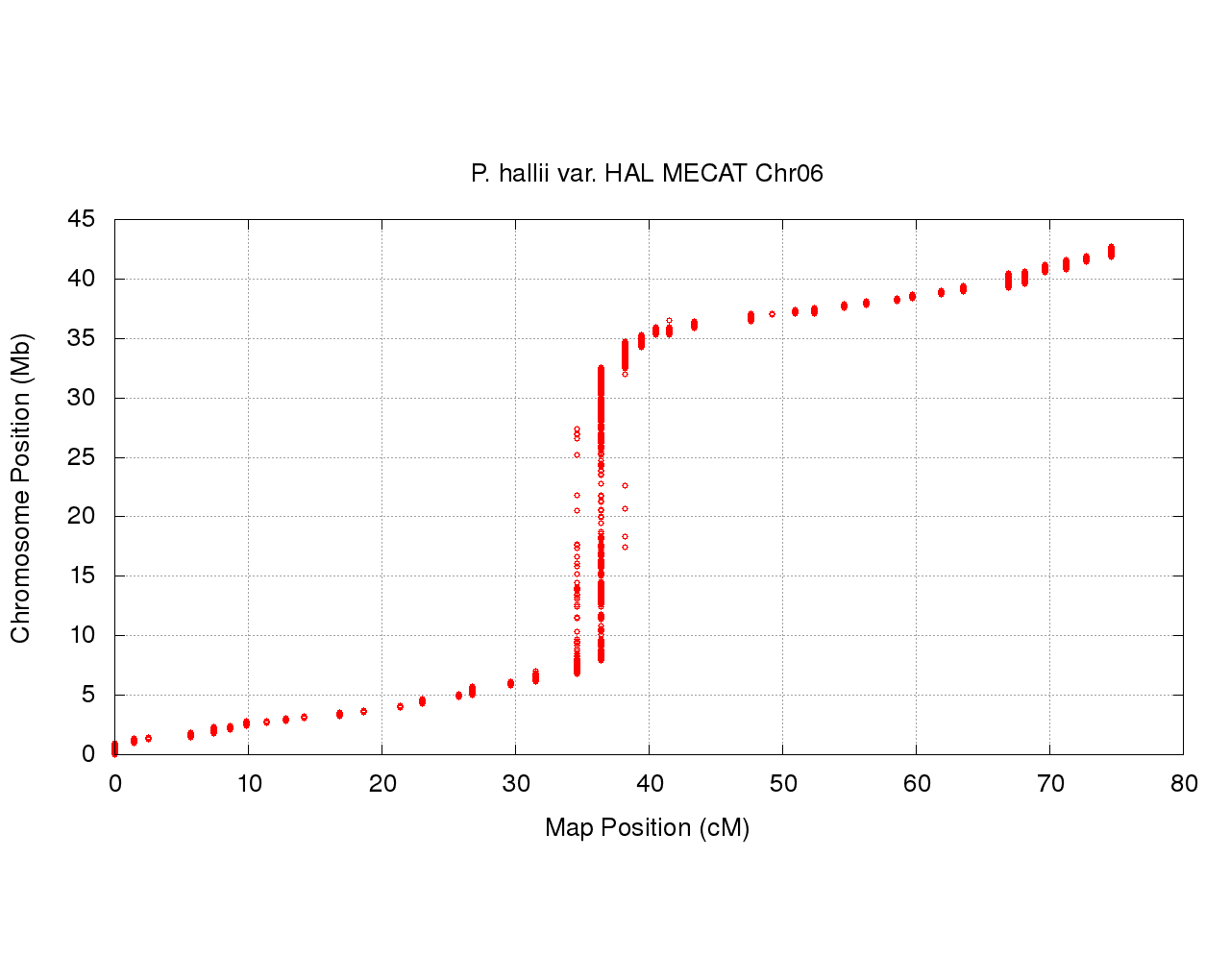 |
| 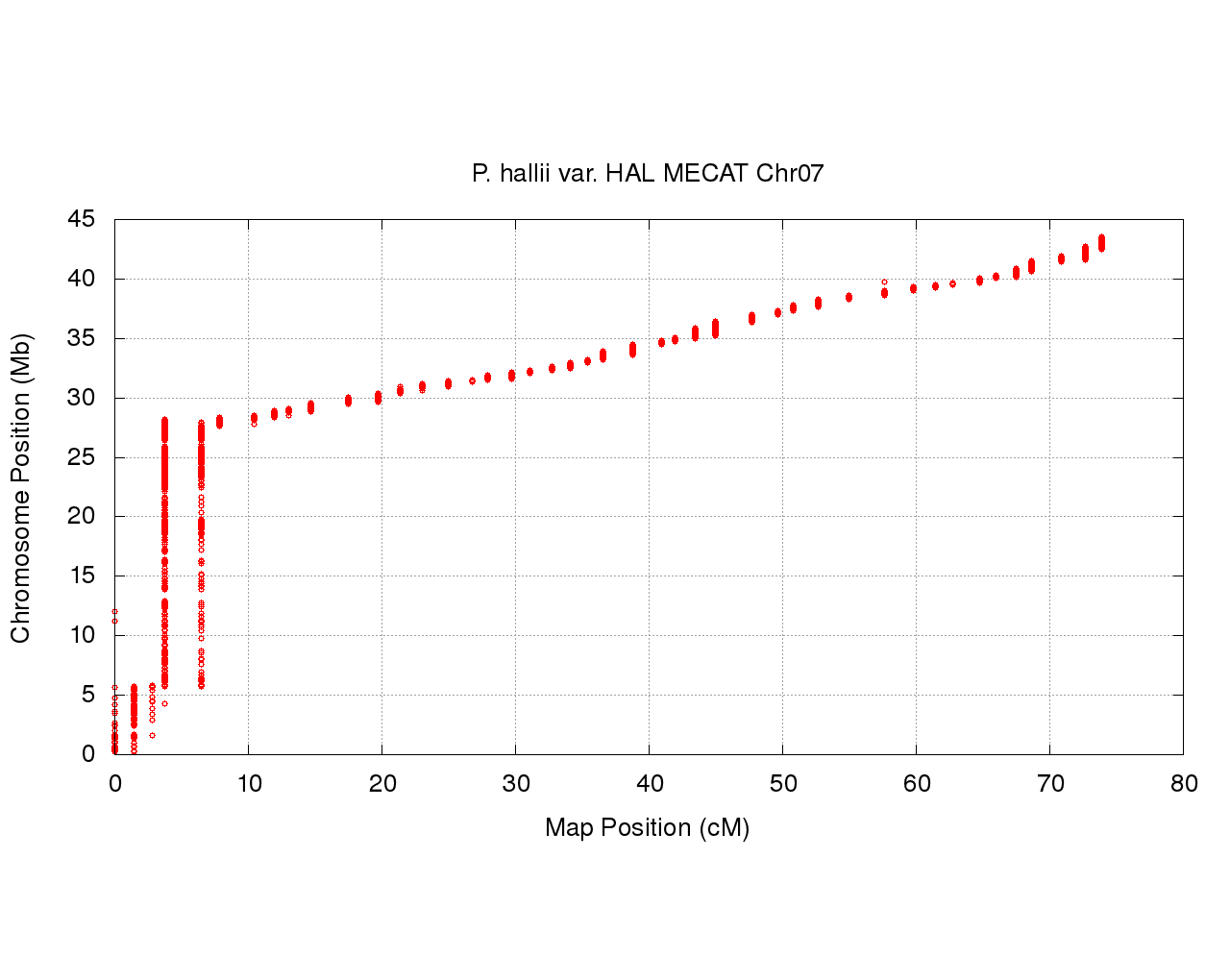 | 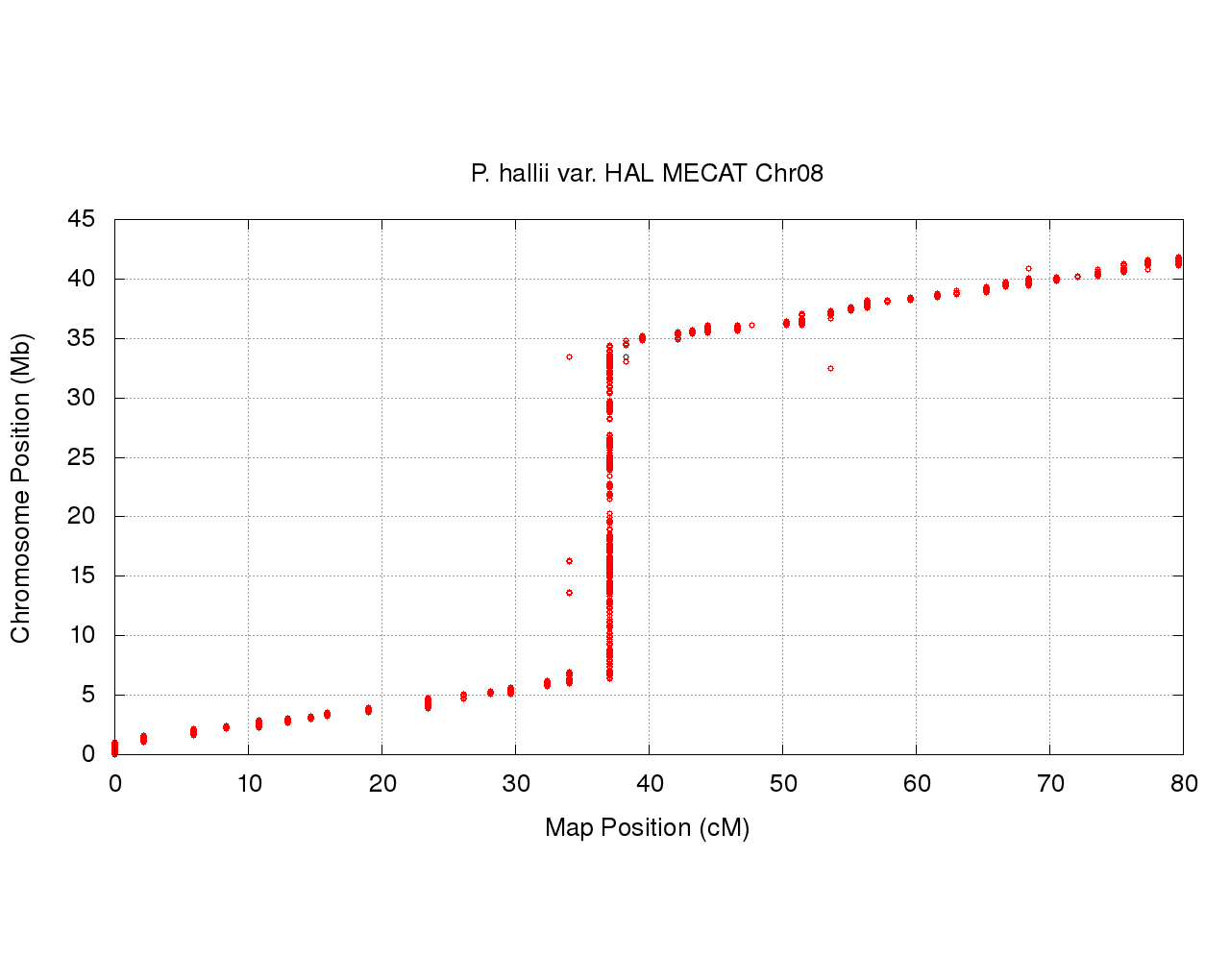 | 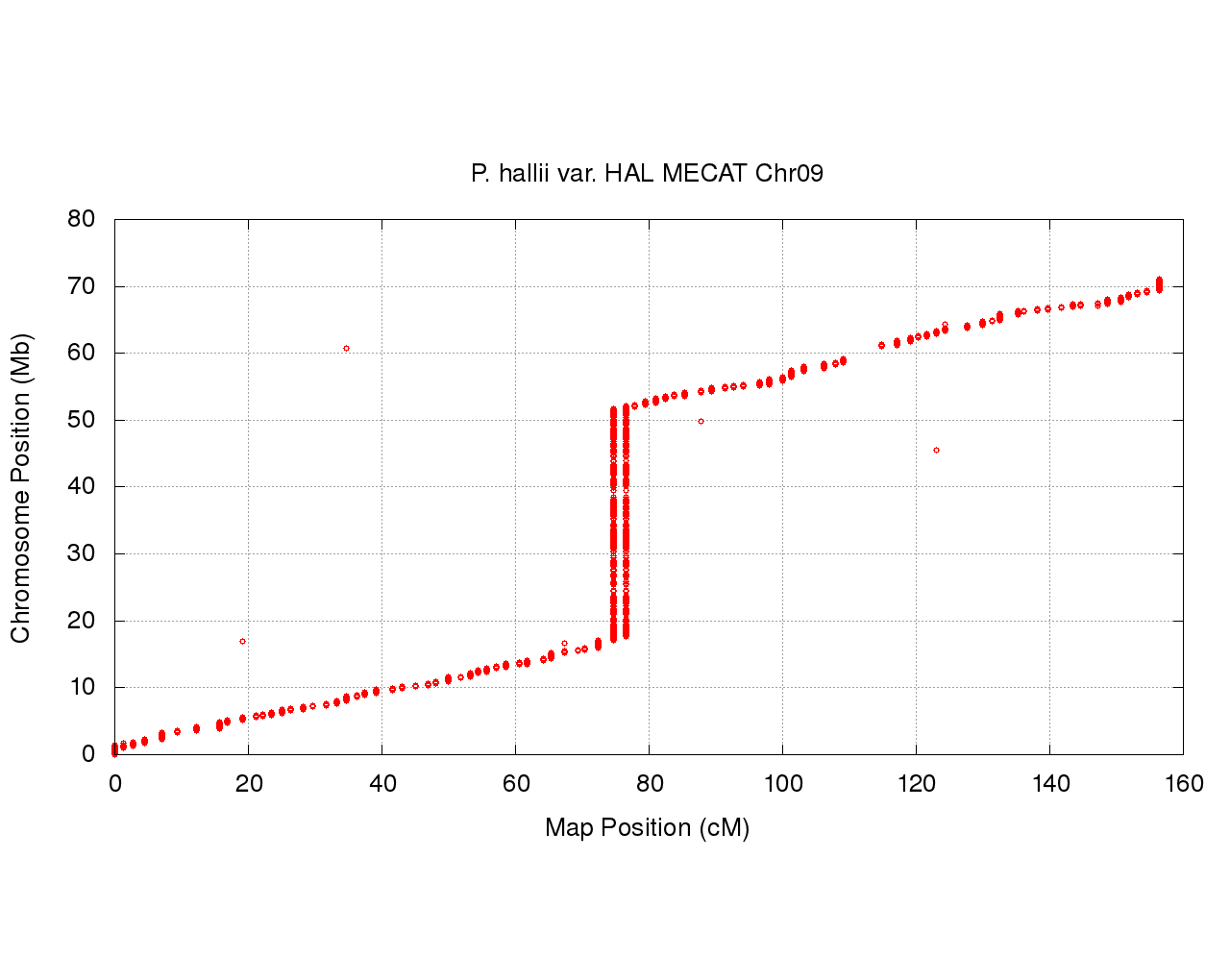 |
| 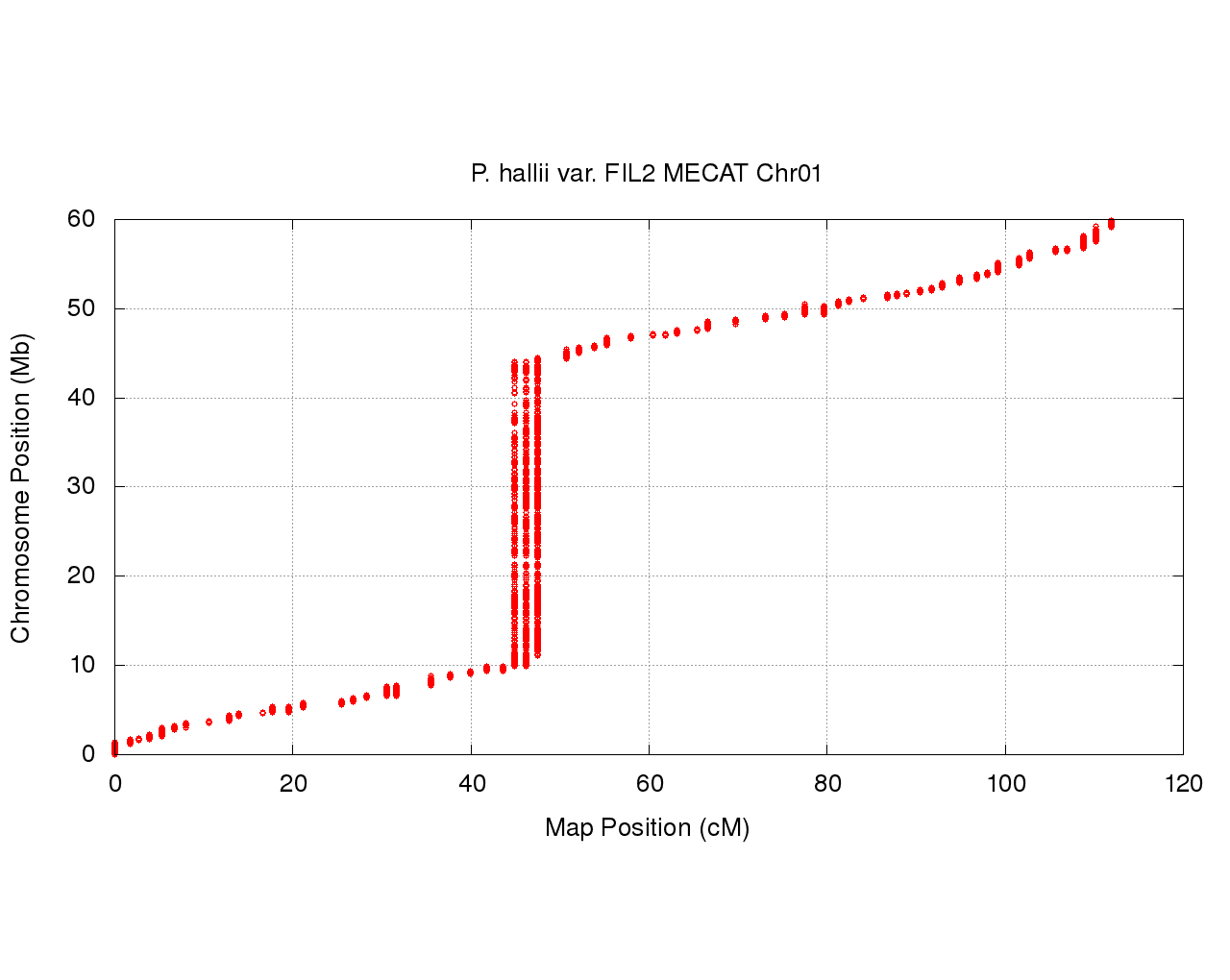 | 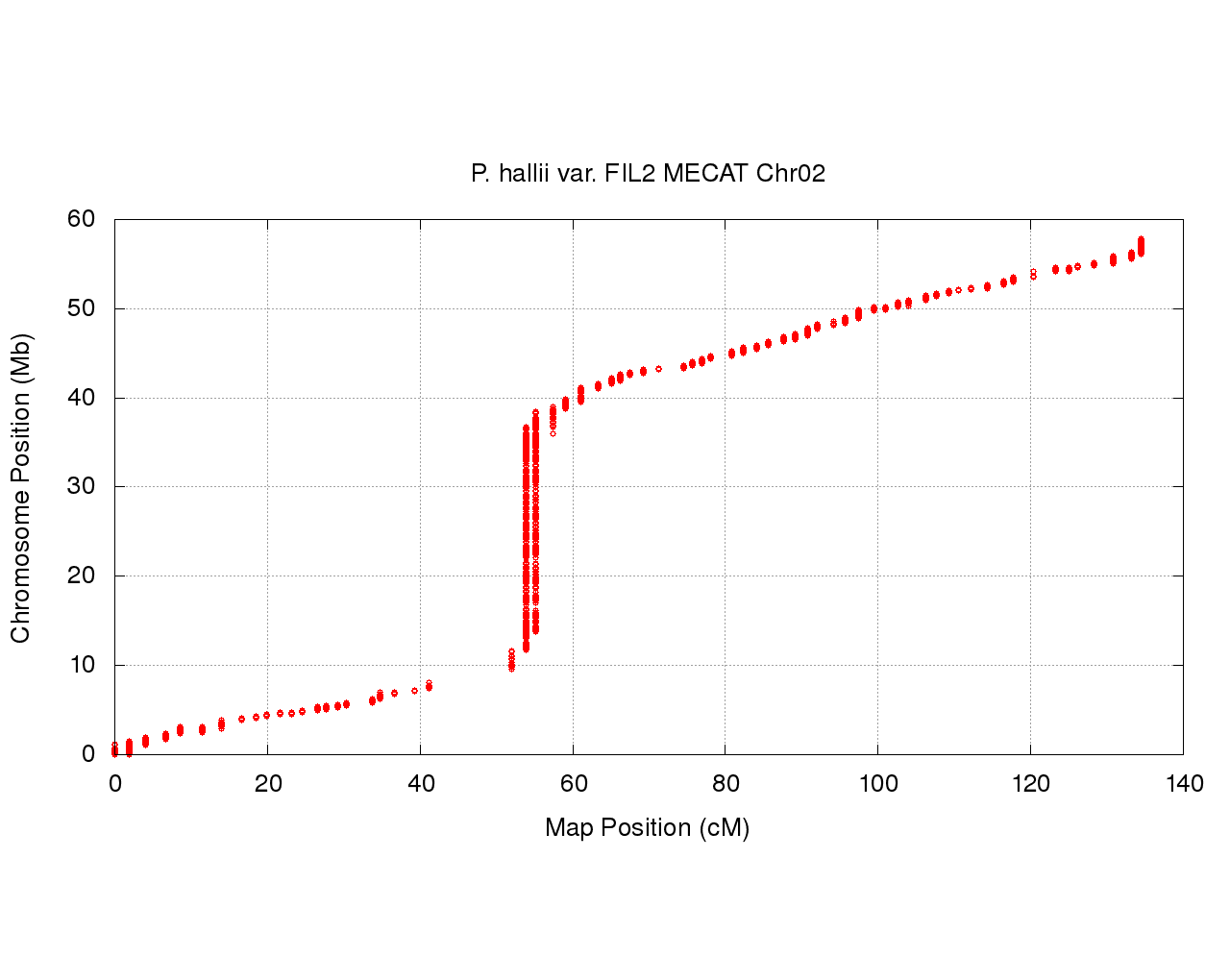 | 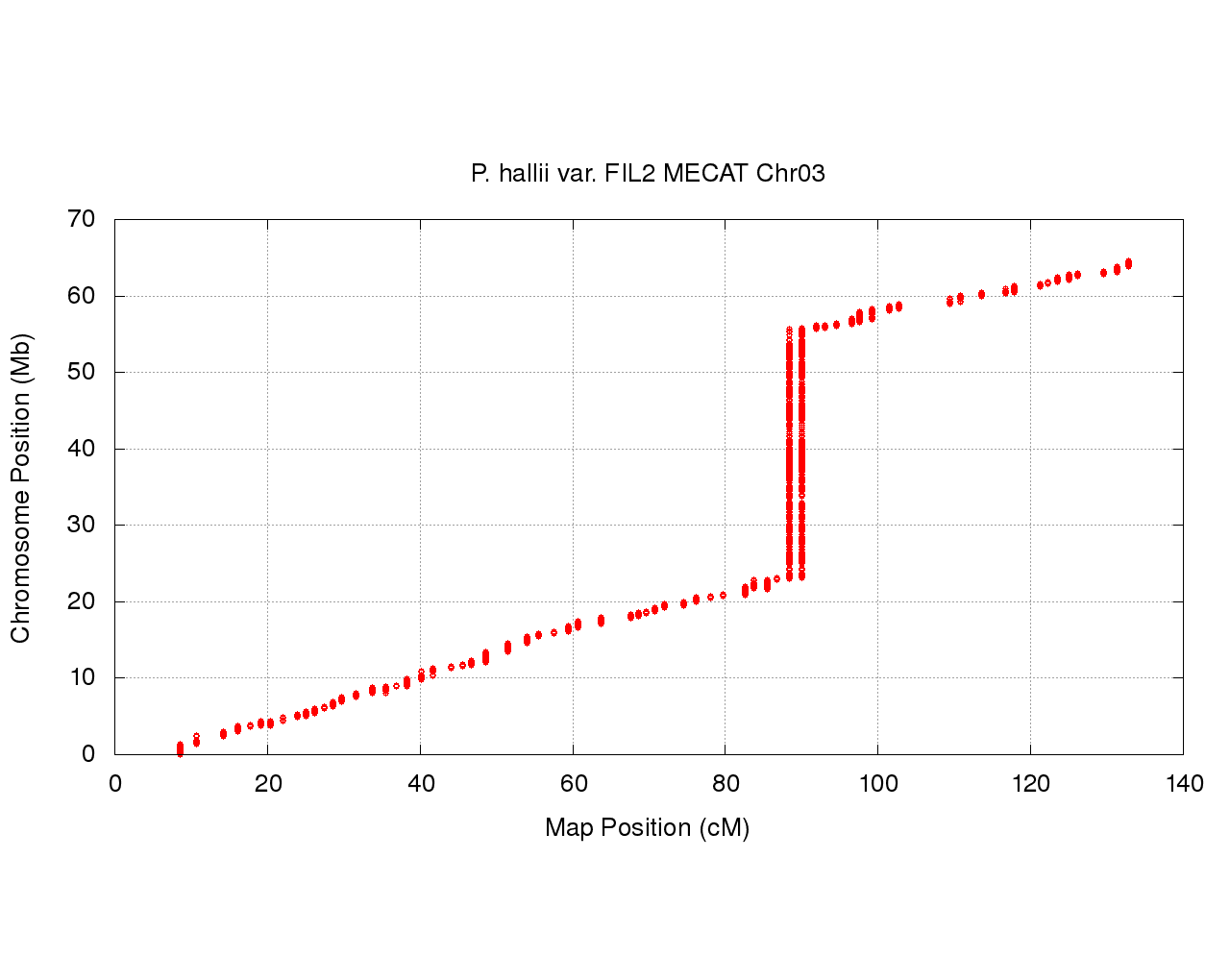 |
| 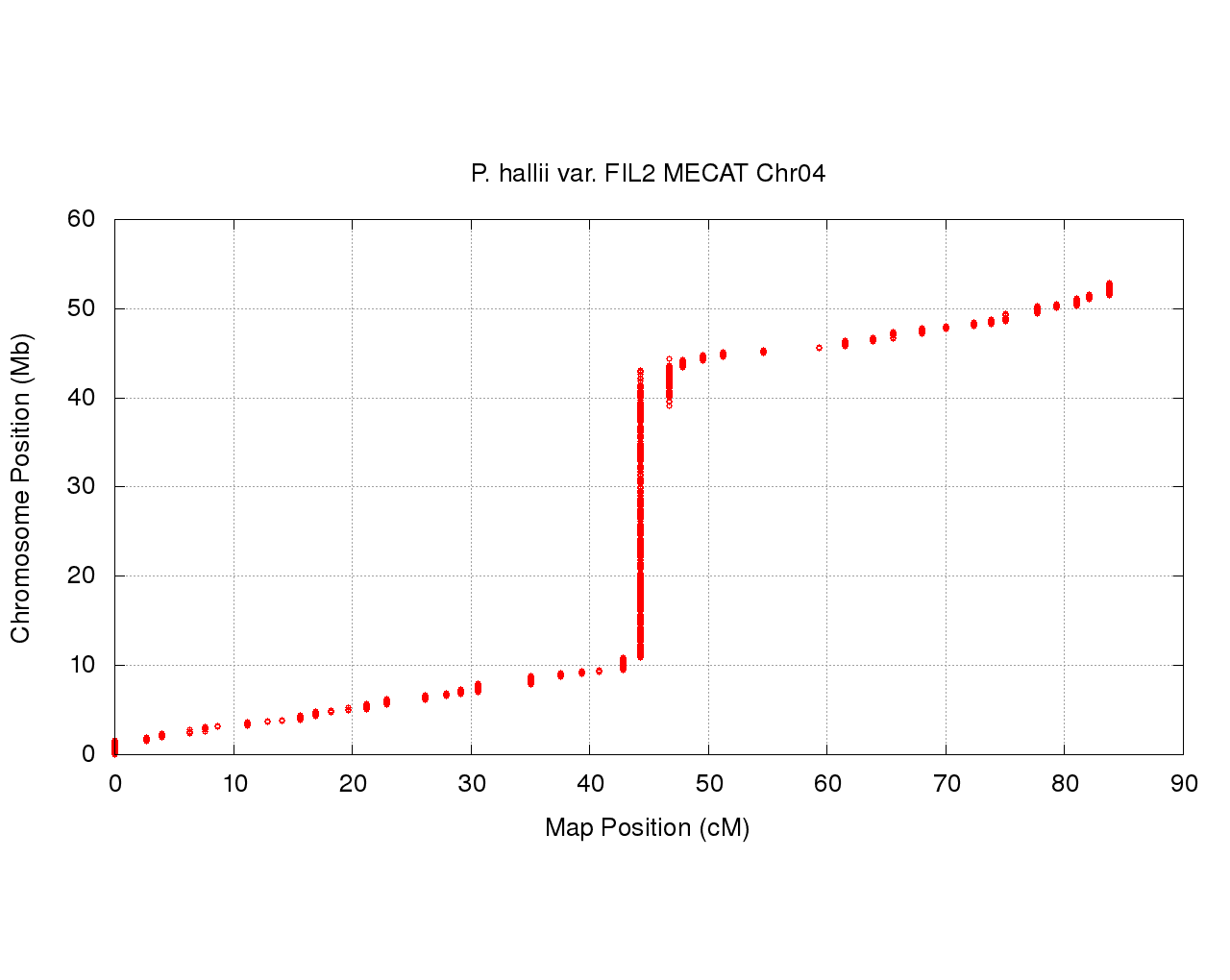 | 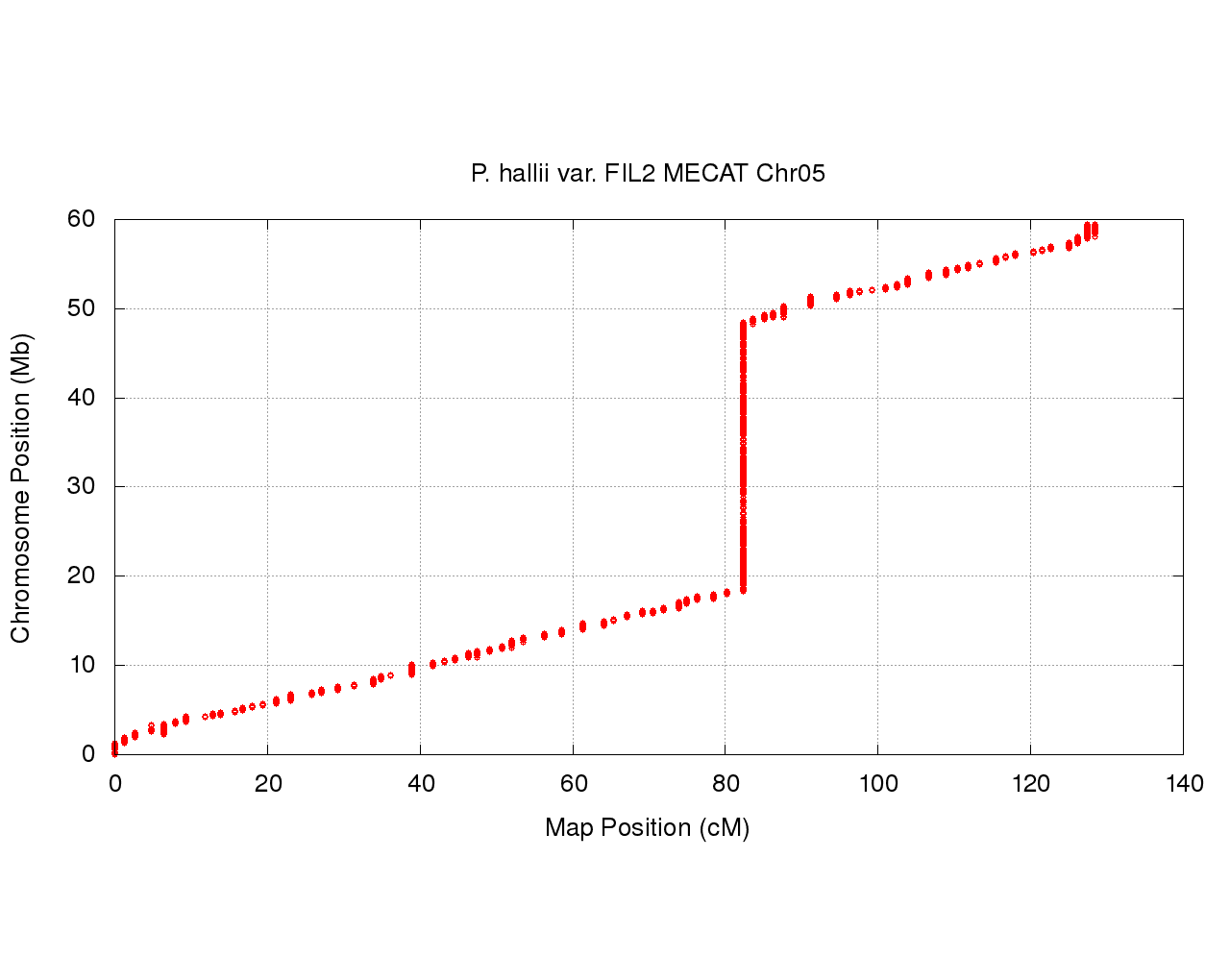 | 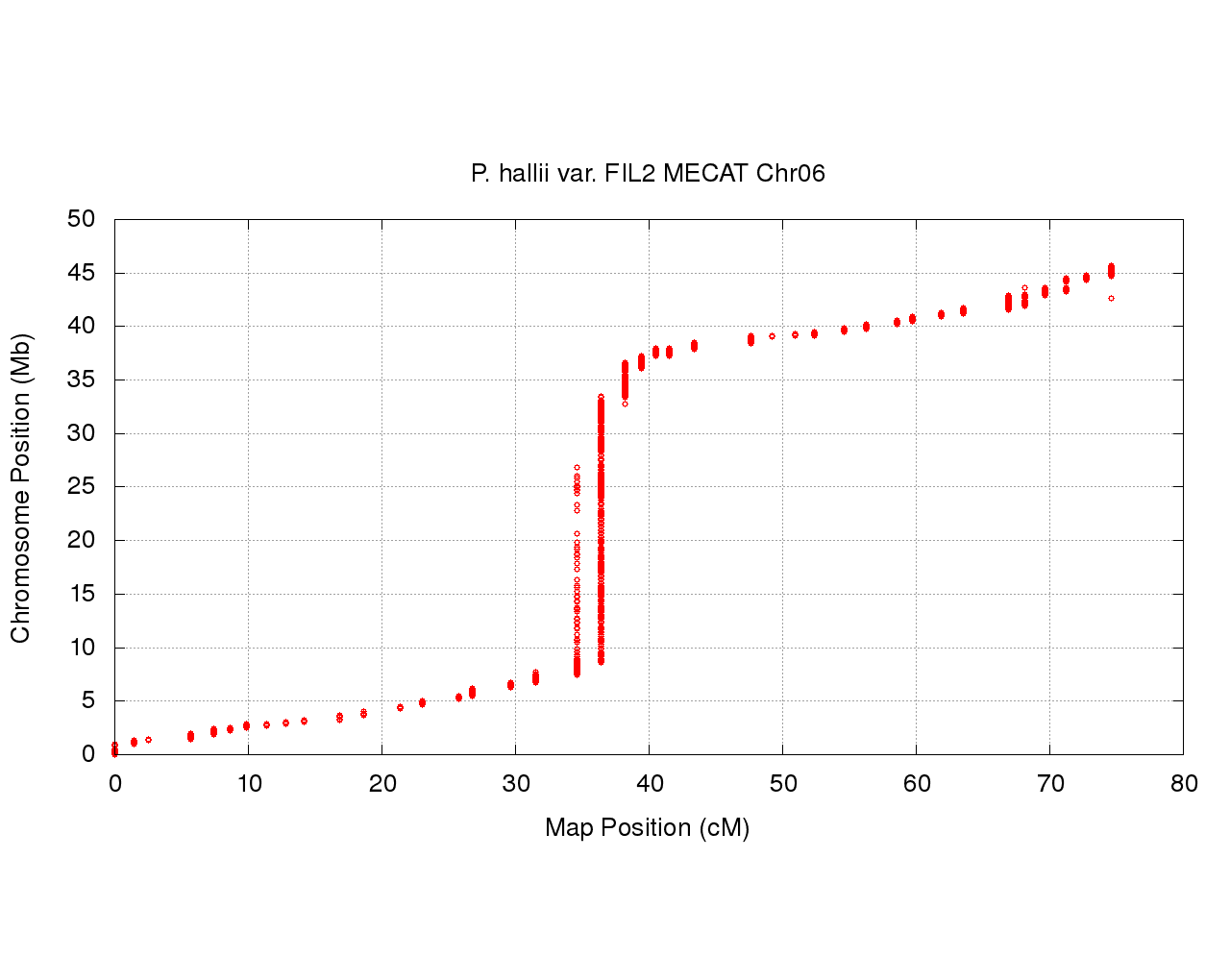 |
| 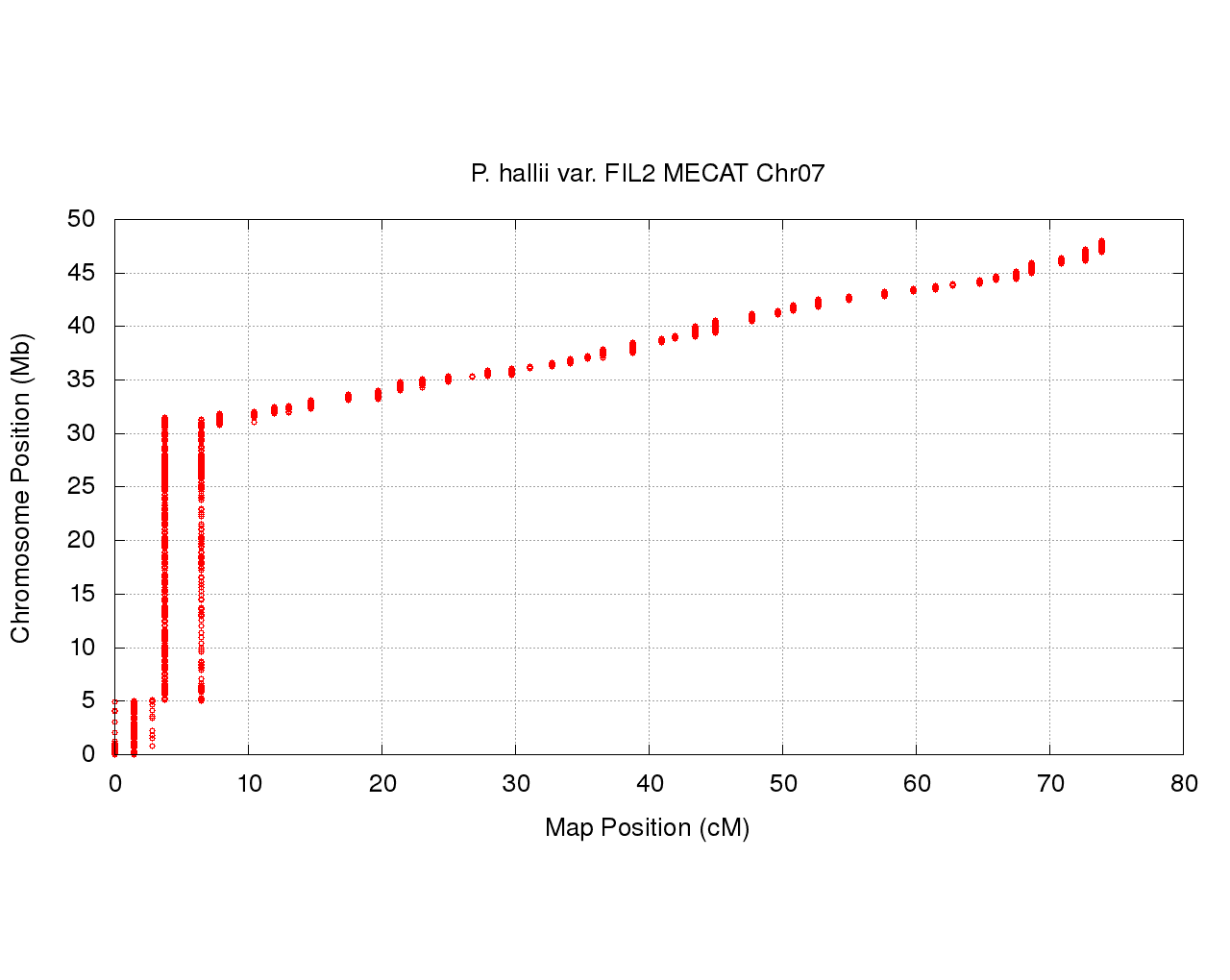 | 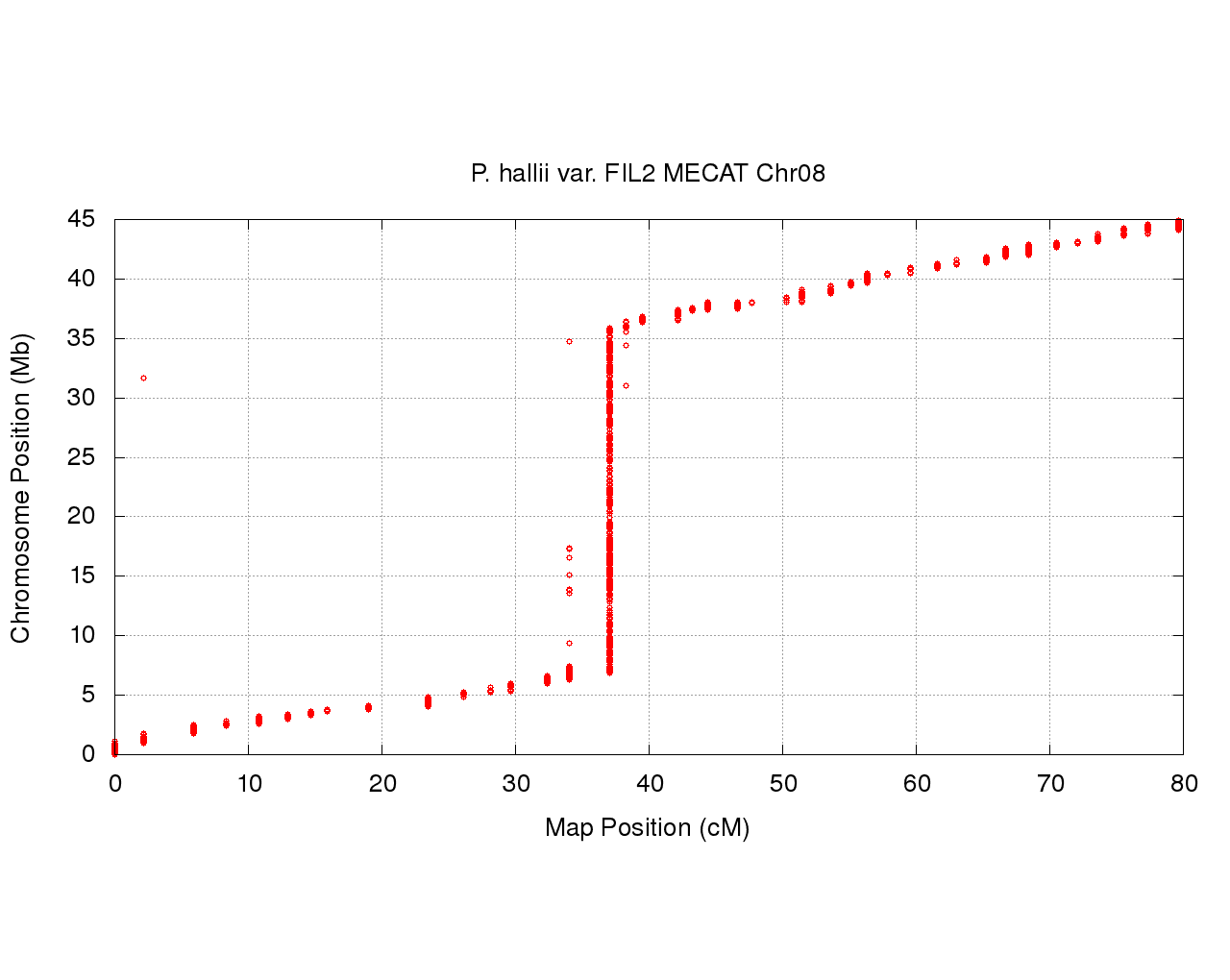 | 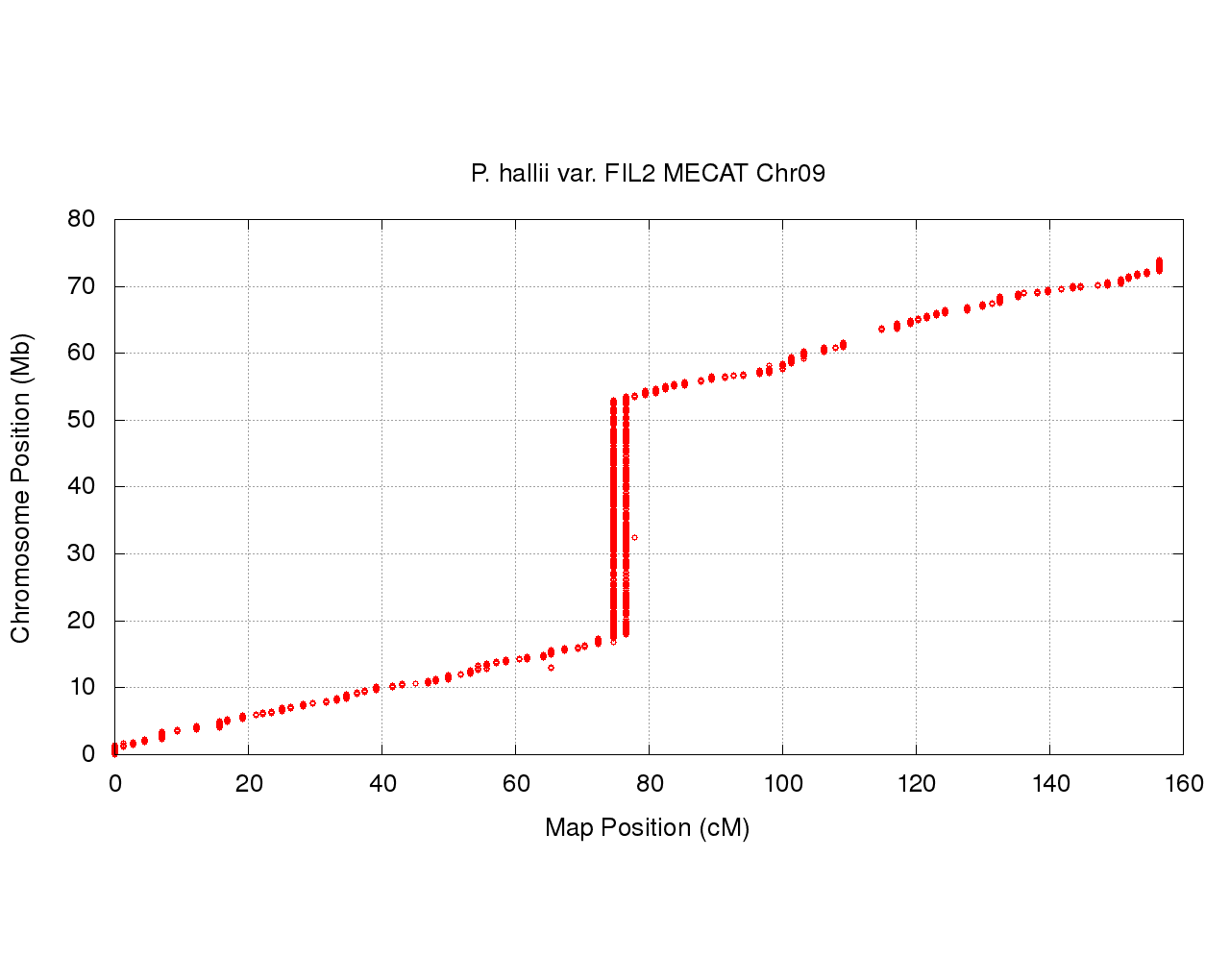 |

**Supplementary Figure 3.** The genetic map was used to order and orient contigs when necessary. The physical position (y-axis) and genetic mapping positions (x-axis) are presented for these markers for HAL2 and FIL2 for each chromosome.

**Supplementary Figure 4.** Comparative sequence ortientation between HAL2 and FIL2 (top) and HAL2 and *S. bicolor* (bottom). Here, genomes are notated as their Phytozome genome IDs; HAL2 = PhalliiHAL, FIL2 = Phallii, *S. bicolor* = Sbicolor. Each point represents an orthologous gene mapping between the two genomes. The axes are gene order between the two genomes and dashed lines indicated the boundaries between chromosomes. Different colors represent different collinear blocks. *S. bicolor* chromosome IDs do not match those of P. hallii. Therefore, the Chr03/08 duplication in P. hallii is on Chr05/08 in *S. bicolor*. Data presented herein can be found in Supplementary Data 2.

**Supplementary Figure 5.** Physiological GxE of HAL2 and FIL2 in the re-water experiment. Means ± standard errors of the mean are presented for each genotype and treatment combination. Experimental n varies by treatment and genotype: FIL2-drought (dry) = 12, FIL2-recovery (wet) = 11, HAL2-drought (dry) = 10, HAL2-recovery (wet) = 5.


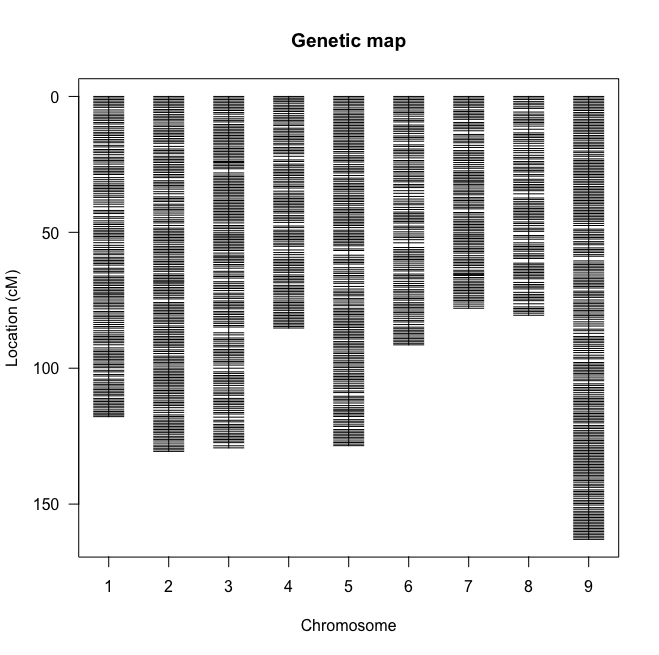


**Supplementary Figure 6.** Graphical depiction of the genetic map. Each horizontal line indicates the presence of a marker for a given mapping position (y-axis) and chromosome (x-axis).

**Supplementary Figure 7.** The degree of pairwise segregation distortion between all pairs of markers in the genetic map. White points are not significantly distorted. *χ* ^2^ statistics with significant *P*-values (grey: -log_10_ *P*-value >1; orange: -log_10_ *P*-value > 5) are colored accordingly.

**Supplementary Figure 8.** Estimates of genotyping error in the F_2_ genetic map. Since the -log likelihood continues to increase to the minimum error probability tested and the map length never experiences a substantial expansion, we estimate the true genotyping error as ≤ 1x10^-6^. This conclusion is bolstered by visual inspection of relative map expansion – as the assumed genotyping error is set lower than the true genotyping error, we expect a dramatic map expansion. This is because genotyping errors will be interpreted as true crossover events, increasing the total mapping length (centimorgans, cM) of a chromosome.

**Supplementary Table 1. Genome release assembly statistics.**

| **FIL2 v3.0** | **Sequencing Platform** | **Average Read/**  **Insert Size** | **Read Number** | **Coverage (x)** |
| --- | --- | --- | --- | --- |
|  | Illumina | 500 | 328,922,392 | 92.10 |
|  | PACBIO | 9,558^*^ | 4,520,785 | 95.87 |
|  | Moleculo |  | 704,618 | 6.9 |
| **Total** |  | N/A | 334,217,795 | 194.87 |

| **HAL2 v2.0** |  |  |  |  |
| --- | --- | --- | --- | --- |
|  | Illumina | 800 | 296,294,162 | 152.3 |
|  | PACBIO | 11,864^*^ | 3,280,170 | 89.53 |
| **Total** |  | N/A | 299,574,332 | 214.83 |

Sequencing platform, effort and coverage are reported for reads used to assemble the HAL2 and FIL2 chromosomal genome sequences. See tables S2-4 for details about contig length coverage. *Indicated median PACBIO insert length.

**Supplementary Table 2. PACBIO coverage statistics.**

|  | **FIL2 (v3)** |  |  |  | **HAL2 (v2)** |  |  |  |
| --- | --- | --- | --- | --- | --- | --- | --- | --- |
| **Min read length** | **Number of Reads** | **Total sequence (Mbp)** | **Mean Read Length (bp)** | **Coverage (x)** | **Number of Reads** | **Total sequence (Mbp)** | **Mean Read Length (bp)** | **Coverage (x)** |
| 0 | 4,520,785 | 47,937 | 9,558 | 95.87 | 3,280,170 | 44,7634 | 11,864 | 89.53 |
| 1,000 | 4,384,573 | 47,853 | 9,860 | 95.71 | 3,141,480 | 44,688 | 12,448 | 89.38 |
| 2,000 | 4,170,989 | 47,531 | 10,342 | 95.06 | 2,988,637 | 44,459 | 13,110 | 88.92 |
| 3,000 | 3,935,519 | 46,941 | 10,896 | 93.88 | 2,836,221 | 44,078 | 13,789 | 88.16 |
| 4,000 | 3,689,695 | 46,080 | 11,501 | 92.16 | 2,687,421 | 43,558 | 14,471 | 87.12 |
| 5,000 | 3,437,280 | 44,944 | 12,150 | 89.89 | 2,544,871 | 42,9167 | 15,147 | 85.83 |
| 6,000 | 3,179,073 | 43,523 | 12,836 | 87.05 | 2,405,559 | 42,150 | 15,825 | 84.30 |
| 7,000 | 2,912,926 | 41,792 | 13,557 | 83.58 | 2,268,548 | 41,260 | 16,510 | 82.52 |
| 8,000 | 2,644,572 | 39,781 | 14,275 | 79.56 | 2,133,331 | 40,246 | 17,205 | 80.49 |
| 9,000 | 2,392,808 | 37,643 | 14,917 | 75.29 | 2,000,426 | 39,117 | 17,903 | 78.23 |
| 10,000 | 2,161,018 | 35,443 | 15,492 | 70.89 | 1,870,511 | 37,883 | 18,597 | 75.77 |
| 11,000 | 1,946,466 | 33,191 | 16,030 | 66.38 | 1,745,283 | 36,569 | 19,286 | 73.14 |
| 12,000 | 1,747,479 | 30,904 | 16,554 | 61.81 | 1,623,791 | 35,172 | 19,972 | 70.34 |
| 13,000 | 1,559,521 | 28,556 | 17,097 | 57.11 | 1,506,841 | 33,711 | 20,658 | 67.42 |
| 14,000 | 1,374,551 | 26,059 | 17,691 | 52.12 | 1,394,829 | 32,199 | 21,347 | 64.40 |
| 15,000 | 1,179,953 | 23,236 | 18,407 | 46.47 | 1,287,699 | 30,646 | 22,034 | 61.29 |
| 16,000 | 979,146 | 20,124 | 19,276 | 40.25 | 1,185,139 | 29,057 | 22,727 | 58.11 |
| 17,000 | 795,831 | 17,102 | 20,243 | 34.20 | 1,086,404 | 27,428 | 23,437 | 54.86 |
| 18,000 | 643,736 | 14,443 | 21,229 | 28.89 | 991,024 | 25,759 | 24,176 | 51.52 |
| 19,000 | 519,700 | 12,151 | 22,219 | 24.30 | 898,363 | 24,045 | 24,938 | 48.09 |

For each set of reads with length at least the number specified in the first column, we record the number and coverage of PacBio data. Data are presented as four columns for each genome.

**Supplementary Table 3. Mecat assembly statistics.**

|  | **FIL2** |  |  |  |  | **HAL2** |  |  |
| --- | --- | --- | --- | --- | --- | --- | --- | --- |
| **Min.**  **Scaffold**  **Length** | **n. scaffolds** | **n. contigs** | **scaffold size (kbp)** | **% Non-gap Basepairs** | **n. scaffolds** | **n. contigs** | **scaffold size (kbp)** | **% Non-gap Basepairs** |
| 5 Mb | 2 | 2 | 12,966 | 100.00% | 32 | 32 | 317,067 | 100.00% |
| 2.5 Mb | 13 | 13 | 53,464 | 100.00% | 61 | 61 | 424,756, | 100.00% |
| 1 Mb | 105 | 105 | 185,346 | 100.00% | 86 | 86 | 466,896 | 100.00% |
| 500 Kb | 298 | 298 | 320,229 | 100.00% | 101 | 101 | 477,935 | 100.00% |
| 250 Kb | 622 | 622 | 436,322 | 100.00% | 113 | 113 | 482,643 | 100.00% |
| 100 Kb | 1,059 | 1,059 | 509,666 | 100.00% | 127 | 127 | 484,910 | 100.00% |
| 50 Kb | 1,328 | 1,328 | 529,653 | 100.00% | 154 | 154 | 486,847 | 100.00% |
| 25 Kb | 1,549 | 1,549 | 537,639 | 100.00% | 185 | 185 | 488,097 | 100.00% |
| 10 Kb | 1,640 | 1,640 | 539,517 | 100.00% | 190 | 190 | 488,202 | 100.00% |
| 5 Kb | 1,644 | 1,644 | 539,549 | 100.00% | 190 | 190 | 488,202 | 100.00% |
| 2.5 Kb | 1,644 | 1,644 | 539,549 | 100.00% | 190 | 190 | 488,202 | 100.00% |
| 1 Kb | 1,644 | 1,644 | 539,549 | 100.00% | 190 | 190 | 488,202 | 100.00% |
| 0 bp | 1,644 | 1,644 | 539,549 | 100.00% | 190 | 190 | 488,202 | 100.00% |

For each set of scaffolds with length at least the number specified in the first column, we record the number and size of Mecat scaffolds and contigs. Data are presented as four columns for each genome.

**Supplementary Table 4. Final assembly statistics**

|  | **FIL v3.0** | **HAL2 v2.0** |
| --- | --- | --- |
| **Scaffold total** | 291 | 43 |
| **Contig total** | 1,027 | 144 |
| **Scaffold sequence total** | 535.9 Mb | 487.5 Mb |
| **Chromosome Sequence** | 500.1 Mb | 483.5 Mb |
| **Contig sequence total** | 528.5 Mb (1.4% gap) | 486.5 Mb (0.2% gap) |
| **Scaffold N/L50** | 5 / 57.9 Mb | 4 / 58.2 Mb |
| **Contig N/L50** | 117 / 1.1 Mb | 15 / 8.3 Mb |

For each released genome version, we present the final composition in terms of number and size of contigs and scaffolds. Data are presented as a single column for each genome.

**Supplementary Table 5. Collinear blocks**

| Block id | Chromosome | HAL2 start | HAL2 end | FIL2 start | FIL2 end | n. genes |
| --- | --- | --- | --- | --- | --- | --- |
| 1 | Chr01 | 14 | 58606 | 10 | 59897 | 3815 |
| 2 | Chr02 | 47 | 57824 | 26 | 57799 | 4247 |
| 5 | Chr03 | 1713 | 1763 | 1499 | 1560 | 10 |
| 7 | Chr03 | 2075 | 58211 | 1771 | 64536 | 3956 |
| 8 | Chr04 | 37 | 18807 | 17 | 19716 | 1428 |
| 9 | Chr04 | 18975 | 50855 | 21046 | 52897 | 1524 |
| 10 | Chr05 | 18 | 59552 | 17 | 59508 | 4758 |
| 11 | Chr06 | 38 | 42738 | 35 | 45706 | 2443 |
| 12 | Chr07 | 442 | 43535 | 142 | 48025 | 2918 |
| 13 | Chr08 | 1974 | 2314 | 2186 | 2602 | 44 |
| 14 | Chr08 | 2518 | 16751 | 2912 | 17422 | 598 |
| 15 | Chr08 | 17132 | 41861 | 17796 | 44927 | 1045 |
| 16 | Chr09 | 31 | 12454 | 17 | 12880 | 1924 |
| 17 | Chr09 | 12461 | 14455 | 13181 | 15009 | 222 |
| 18 | Chr09 | 14502 | 14690 | 12926 | 13127 | 29 |
| 19 | Chr09 | 14704 | 71069 | 15066 | 73895 | 3569 |
|  |  |  |  |  |  |  |
| 4 | Chr03 \| Chr08 | 40\|21 | 1711\|1973 | 8\|11 | 1497\|2185 | 568 |
| 6 | Chr03 \| Chr08 | 1766\|2319 | 2072\|2518 | 1563\|2607 | 1767\|2911 | 84 |
|  |  |  |  |  |  |  |
| 3 | Chr02 & Chr04 | 9197 | 9492 | 20326 | 20594 | 22 |

Coordinates of all 19 unique collinear blocks between HAL2 and FIL2. Single copy, collinear blocks have a single chromosome name, while duplicated block chromosomes are ‘|’ separated and translocated block chromosomes are separated by ‘&’. All positions are in kilobases (kb). ‘n. genes’ indicates the number of HAL2 gene models within orthogroups in each block.

**Supplementary Table 6. QTL scans for leaf water potential in the F_2_**

| Phenotype | Chromosome | Position (cM) | LOD score | *P*-value |
| --- | --- | --- | --- | --- |
| Predawn LWP | 1 | 39.02 | 1.53 | 0.99 |
|  | 2 | 80.69 | 1.15 | 1 |
|  | 3 | 0 | 1.83 | 0.93 |
|  | 4 | 41.5 | 0.72 | 1 |
|  | 5 | 45 | 1.41 | 1 |
|  | 6 | 4.04 | 1.16 | 1 |
|  | 7 | 51.88 | 1.12 | 1 |
|  | 8 | 27.89 | 2.9 | 0.27 |
|  | 9 | 94.77 | 2.23 | 0.7 |
| Midday LWP | 1 | 38 | 1.96 | 0.89 |
|  | 2 | 14.78 | 1.75 | 0.97 |
|  | 3 | 14.41 | 2.6 | 0.44 |
|  | 4 | 38 | 1.86 | 0.93 |
|  | 5 | 122.98 | 0.94 | 1 |
|  | 6 | 18 | 1.59 | 0.99 |
|  | 7 | 40.36 | 0.96 | 1 |
|  | 8 | 38 | 1.31 | 1 |
|  | 9 | 94.77 | 1.74 | 0.97 |

Maximum QTL peaks for predawn and midday leaf water potential (LWP) in the F_2_ mapping population. These results are for the physiology data that was paired with the eQTL RNA extraction. LOD peaks are calculated from one-way QTL scans using the Haley-Knott regression and controlling for the additive covariate of drought treatment. Empirical *P*-values are derived from 1000 permutations of one-way QTL scans using the same parameters.

**Supplementary Table 7. ABO3 expression**

| Genotype | Treatment | ABO3 allele | Mean expression | Standard deviation |
| --- | --- | --- | --- | --- |
| 201 | Dry | FIL | 10.5787128 | 15.0891256 |
| 206 | Dry | FIL | 2.22406968 | 2.0261244 |
| 270 | Dry | FIL | 0.50803156 | 0.38142917 |
| 816 | Dry | FIL | 4.71593803 | 6.23225641 |
| 825 | Dry | FIL | 1.48763151 | 0.49287068 |
| 880 | Dry | FIL | 12.2662585 | 18.4182752 |
| FIL2 | Dry | FIL | 0.69600617 | 0.41420154 |
| 201 | Wet | FIL | 49.4312094 | 55.117956 |
| 206 | Wet | FIL | 9.0580505 | 9.46456089 |
| 270 | Wet | FIL | 0.82632298 | 0.43556883 |
| 816 | Wet | FIL | 1.4790963 | 0.46569097 |
| 825 | Wet | FIL | 6.08236531 | 7.21417887 |
| 880 | Wet | FIL | 5.8694043 | 9.96648684 |
| FIL2 | Wet | FIL | 0.53008755 | 0.31805576 |
| 183 | Dry | HAL | 62.3587649 | 30.0389466 |
| 252 | Dry | HAL | 98.3032905 | 52.5715596 |
| 277 | Dry | HAL | 37.6270605 | 11.1287566 |
| 35 | Dry | HAL | 22.0847002 | 16.0200978 |
| 426 | Dry | HAL | 48.9879278 | 10.8261926 |
| 464 | Dry | HAL | 37.2940158 | 34.0562441 |
| 841 | Dry | HAL | 30.2879466 | 4.34012072 |
| 898 | Dry | HAL | 141.883399 | 43.9826831 |
| HAL2 | Dry | HAL | 119.183729 | 132.309584 |
| 183 | Wet | HAL | 112.598258 | 88.6393078 |
| 252 | Wet | HAL | 72.0103028 | 30.2155925 |
| 277 | Wet | HAL | 200.74491 | 142.551774 |
| 35 | Wet | HAL | 34.3067192 | 35.0184065 |
| 426 | Wet | HAL | 119.367425 | 132.219046 |
| 464 | Wet | HAL | 49.7383531 | 42.7260779 |
| 841 | Wet | HAL | 54.476979 | 29.3447144 |
| 898 | Wet | HAL | 72.5408411 | 43.7802776 |
| HAL2 | Wet | HAL | 108.926466 | 111.134322 |

Summary of RT-qPCR assayed expression of the *ABO3* gene in HAL2, FIL2 and a set of 15 recombinant inbred lines that recombine in proximity to the ABO3 gene. Plants are grown following the RIL drought experiment. The mean and standard deviation of expression are presented for each genotype-by-treatment combination. The allele of ABO3 is also presented for each RIL.

**Supplementary Table 8. Allelic effect in dry-down experiment**

| hotspot | phenotype | treatment | Estimate | SE | LOD | PVE | *P*-value | *χ* ^2^ _df = 12_ | Combined *P* |
| --- | --- | --- | --- | --- | --- | --- | --- | --- | --- |
| 3a | RWC | Diff. | -0.005 | 0.003 | 0.827 | 1.336 | 0.052 | 27.3 | 0.0068 |
| 3a | SPAD | Diff. | 0.076 | 0.19 | 0.035 | 0.057 | 0.688 |  |  |
| 3a | RWC | Dry | 0.004 | 0.002 | 0.668 | 1.073 | 0.081 |  |  |
| 3a | SPAD | Dry | 0.331 | 0.164 | 0.884 | 1.419 | 0.045 |  |  |
| 3a | RWC | Wet | -0.001 | 0.002 | 0.079 | 0.128 | 0.549 |  |  |
| 3a | SPAD | Wet | 0.407 | 0.168 | 1.27 | 2.031 | 0.016 |  |  |
| 3b | RWC | Diff. | 0.002 | 0.003 | 0.078 | 0.128 | 0.55 | 10.8 | 0.54 |
| 3b | SPAD | Diff. | 0.289 | 0.188 | 0.517 | 0.832 | 0.124 |  |  |
| 3b | RWC | Dry | -0.001 | 0.002 | 0.015 | 0.024 | 0.793 |  |  |
| 3b | SPAD | Dry | -0.161 | 0.164 | 0.212 | 0.342 | 0.325 |  |  |
| 3b | RWC | Wet | 0.001 | 0.002 | 0.073 | 0.118 | 0.565 |  |  |
| 3b | SPAD | Wet | 0.128 | 0.169 | 0.125 | 0.202 | 0.45 |  |  |
| 7 | RWC | Diff. | 0.002 | 0.002 | 0.14 | 0.228 | 0.423 | 19.5 | 0.072 |
| 7 | SPAD | Diff. | -0.186 | 0.171 | 0.258 | 0.416 | 0.278 |  |  |
| 7 | RWC | Dry | -0.005 | 0.002 | 1.294 | 2.07 | 0.015 |  |  |
| 7 | SPAD | Dry | 0.007 | 0.15 | 0 | 0.001 | 0.962 |  |  |
| 7 | RWC | Wet | -0.002 | 0.002 | 0.484 | 0.785 | 0.137 |  |  |
| 7 | SPAD | Wet | -0.179 | 0.153 | 0.297 | 0.479 | 0.244 |  |  |

Tests of allelic effects at three trans-eQTL hotspots on six treatment-trait combinations. Estimate and standard error are the effects of replacing the FIL2 allele with the HAL2 allele. LOD score and percent variance explained (PVE) are for a model testing between a NULL model and one with the alleles at a given QTL. The *P*-value is the *F*-statistic significance of this model comparison. The last two columns give the Fisher’s Combined test of significance for each locus across all six treatment-trait combinations. This is a *χ*^2^-statistic with a degree of freedom of twice the number of *P*-values. The *P*-value of this combined test is given in the last column.

**Supplementary References**

1. Xiao, C.-L. *et al.* MECAT: fast mapping, error correction, and <i>de novo</i> assembly for single-molecule sequencing reads. *Nature Methods* **14,** 1072 (2017).

2. Chin, C.-S. *et al.* Nonhybrid, finished microbial genome assemblies from long-read SMRT sequencing data. *Nature Methods* **10,** 563–569 (2013).

3. Kent, W. J. BLAT--the BLAST-like alignment tool. *Genome Research* **12,** 656–664 (2002).

4. Li, H. & Durbin, R. Fast and accurate short read alignment with Burrows-Wheeler transform. *Bioinformatics* **25,** 1754–1760 (2009).

5. McKenna, A. *et al.* The Genome Analysis Toolkit: a MapReduce framework for analyzing next-generation DNA sequencing data. *Genome Research* **20,** 1297–1303 (2010).
