# Supplementary material for: The genomic landscape of molecular responses to natural drought stress in Panicum hallii
Source: Nat Commun. 2018 Dec 6;9:5213. doi: 10.1038/s41467-018-07669-x (PMC6283873; doi:10.1038/s41467-018-07669-x)
Supplement: Supplementary file 3 — Description of Additional Supplementary Files [file 41467_2018_7669_MOESM3_ESM.pdf]

Description of Additional Supplementary Files

Supplementary Data 1
Description: Population-genetic library metadata, including library sequencing identifiers, public repository information and sequencing effort data for each of the re-sequenced genotypes used in the population genetic analysis.

Supplementary Data 2
Description: Demographic modeling data. including effective population size (Ne) and relative cross coalescence (cc) along with the time interval and bootstrap confidence interval.

Supplementary Data 3
Description: List of 1:1 orthologs, including the list of reciprocal best hit (RBH) orthologs, where the highest scoring HAL2-FIL2 protein blat hit matches the gene model of the FIL2-HAL2 protein blat hit. As a naming convention, these gene pairs are collapsed to their HAL2 gene identifier.

Supplementary Data 4
Description: Categorization of orthogroups, including a list of genes, their assigned orthogroups and categorization of orthogroups following Table 1.

Supplementary Data 5
Description: List of private gene models including a list of gene IDs that are private to either HAL2 or FIL2 annotations.

Supplementary Data 6
Description: Summary of presence-absence variation. This file contains the list of genes that display presence-absence variation. Categories follow Table 1.

Supplementary Data 7
Description: eQTL library metadata. This file contains library sequencing identifiers, public repository information and sequencing effort data for each of the total RNA-sequenced F2, F1 and parental libraries in our drought/recovery eQTL experiment.

Supplementary Data 8
Description: eQTL statistical output. This file contains statistical output from the eQTL analysis, including effect direction, positional categorization and statistical support.

Supplementary Data 9
Description: CDS variants by gene. This file contains the gene-by-gene summary output from SNPeff and gene-by-gene counts of total insertion/deletion (INDEL) polymorphism. We categorized both INDELs and SNPs into groups based on their predicted effect.

Supplementary Data 10
Description: Transcription factor binding affinity. This file the gene-by-gene affinity of each of 35 transcription factors to the 2kb upstream region of each gene. For each TF, the affinity was scored by a discrete count.
