# Supplementary material for: The genomic landscape of molecular responses to natural drought stress in Panicum hallii
Source: Nat Commun. 2018 Dec 6;9:5213. doi: 10.1038/s41467-018-07669-x (PMC6283873; doi:10.1038/s41467-018-07669-x)
Supplement: Supplementary file 14 — Reporting Summary [file 41467_2018_7669_MOESM14_ESM.pdf]

nature research

Corresponding author(s): John Lovell, Tom Juenger, Jeremy Schmutz

Reporting Summary

Nature Research wishes to improve the reproducibility of the work that we publish. This form provides structure for consistency and transparency in reporting. For further information on Nature Research policies, see Authors & Referees and the Editorial Policy Checklist.

Statistical parameters

When statistical analyses are reported, confirm that the following items are present in the relevant location (e.g. figure legend, table legend, main text, or Methods section).

n/a	Confirmed	
☐	☒	The exact sample size (*n*) for each experimental group/condition, given as a discrete number and unit of measurement
☐	☒	An indication of whether measurements were taken from distinct samples or whether the same sample was measured repeatedly
☐	☒	The statistical test(s) used AND whether they are one- or two-sided<br>*Only common tests should be described solely by name; describe more complex techniques in the Methods section.*
☐	☒	A description of all covariates tested
☐	☒	A description of any assumptions or corrections, such as tests of normality and adjustment for multiple comparisons
☐	☒	A full description of the statistics including central tendency (e.g. means) or other basic estimates (e.g. regression coefficient) AND variation (e.g. standard deviation) or associated estimates of uncertainty (e.g. confidence intervals)
☐	☒	For null hypothesis testing, the test statistic (e.g. *F*, *t*, *r*) with confidence intervals, effect sizes, degrees of freedom and *P* value noted<br>*Give P values as exact values whenever suitable.*
☒	☐	For Bayesian analysis, information on the choice of priors and Markov chain Monte Carlo settings
☐	☒	For hierarchical and complex designs, identification of the appropriate level for tests and full reporting of outcomes
☐	☒	Estimates of effect sizes (e.g. Cohen's *d*, Pearson's *r*), indicating how they were calculated
☐	☒	Clearly defined error bars<br>*State explicitly what error bars represent (e.g. SD, SE, CI)*

Our web collection on statistics for biologists may be useful.

Software and code

Policy information about availability of computer code

Data collection	All bioinformatics programs used in this study are open source. They include bioinformatics software (samtools, EXONERATE, blat, BLAST, diamond, MCScanX, star, ht-seq, bwa-mem, minimap2)
Data analysis	All statistical analysis programs used in this study are open source, implemented in the R environment for statistical computing. Code for qtl analysis pipeline has been deposited on github.com/jtlovell/qtlTools.

For manuscripts utilizing custom algorithms or software that are central to the research but not yet described in published literature, software must be made available to editors/reviewers upon request. We strongly encourage code deposition in a community repository (e.g. GitHub). See the Nature Research guidelines for submitting code & software for further information.

Data

Policy information about availability of data

All manuscripts must include a data availability statement. This statement should provide the following information, where applicable:

- Accession codes, unique identifiers, or web links for publicly available datasets
- A list of figures that have associated raw data
- A description of any restrictions on data availability

All RNA and resequencing reads have been deposited in the NCBI short read archive (https://www.ncbi.nlm.nih.gov/sra). Bioprojects, sample IDs and metadata can

be found in Supplementary Data 1 and 7. Both genome assemblies and annotations are available through phytozome (https://phytozome.jgi.doe.gov). The assemblies have also been deposited on genbank (https://www.ncbi.nlm.nih.gov/genbank) under BioProjects PRJNA251785[https://www.ncbi.nlm.nih.gov/bioproject/251785] (HAL2) and PRJNA250527[https://www.ncbi.nlm.nih.gov/bioproject/250527] (FIL2). All statistical, QTL mapping and visualization functions were implemented in R 3.4.3 and have been compiled into an R package stored on github.com/jtlovell/qtlTools. Additional supporting data is provided as Supplementary Data. Details are provided in the Description of Additional Supplementary Files. The source data underlying Figures 1A-B, 2A-D, 3B-C and Supplementary Figures 3-7 are provided as a Source Data File. Source data for Figure 3A,D and Supplementary Figures 1-2 are found in Supplementary Data 7 and Supplementary Data 2, respectively.

Field-specific reporting

Please select the best fit for your research. If you are not sure, read the appropriate sections before making your selection.

☐ Life sciences ☐ Behavioural & social sciences ☒ Ecological, evolutionary & environmental sciences

For a reference copy of the document with all sections, see nature.com/authors/policies/ReportingSummary-flat.pdf

Ecological, evolutionary & environmental sciences study design

All studies must disclose on these points even when the disclosure is negative.

Study description	We assembled and annotated two complete genomes from two unique genotypes. 243 F2 lines were grown under drought and half the plants were re-watered. This drought treatment is the primary covariate for all analyses.
Research sample	Natural collections and experimental mapping populations of the perennial C4 grass, Panicum hallii.
Sampling strategy	Sample size power calculations were not performed. Instead, from previous work, we knew that 243 F2 genotypes would be sufficient to define QTL of moderate effect.
Data collection	The physiological data was collected manually (headed by T. Juenger and J. Bonnette) , while sequencing data collection followed standard sequencing procedures.
Timing and spatial scale	Field collections were conducted at a single site. Drought treatment plants were harvested on 5-July 2013, while "recovery" treatment plants received 4 L of water on 7-July 2013 and were harvested on 8-July 2013. For each plant, we measured midday leaf water potential (LWP, Ψleaf) with a Scholander-type pressure bomb (PMS Instruments, model 1000) between 11:00 and 13:00. All plants reached anthesis by 5-July 2013.
Data exclusions	Contaminated samples were not included in the analysis. These were determined via clustering.
Reproducibility	While each experiment was conducted only once, the RIL was explicitly a follow up on the results of the F2.
Randomization	Planting design were randomized split-plots.
Blinding	The plant IDs do not indicate the genotype or treatment of the plant, except in a database. However, it is not feasible to do plant physiology without looking at the plant. Blinding in this manner was impossible.

Did the study involve field work? ☒ Yes ☐ No

Field work, collection and transport

Field conditions	Plants were subjected to ambient conditions, planted directly in native soil. Additional watering at establishment was provided as needed. Recovery treatment plants were irrigated with 4L of water 24h prior to measurements.
Location	Brackenridge Field Laboratory (Austin, TX, USA; 30.284,-97.778) and Ladybird Johnson Wildflower Center (Austin, TX, USA; 30.19° N, 97.87° W).
Access and import/export	All seed collections were made with the permissions of at least one of the following: The Ladybird Johnson Wildflower Center, The Brackenridge Field Laboratory, The Nature Conservancy of Texas, The State Parks of Texas, The Kika de la Garza Plant Materials Center, The University of Arizona and Arizona State University Herbaria, The University of Texas Ecolabs Properties, USDA GRIN Stock Center and Angela Safranek (USFS).
Disturbance	Collection of seeds was very limited in scope and conducted carefully as to not affect native vegetation. All other experimentation was conducted in field sites that had been used previously for field trials.

Reporting for specific materials, systems and methods

Materials & experimental systems

n/a	Involved in the study
☐	☒ Unique biological materials
☒	☐ Antibodies
☒	☐ Eukaryotic cell lines
☒	☐ Palaeontology
☒	☐ Animals and other organisms
☒	☐ Human research participants

Methods

n/a	Involved in the study
☒	☐ ChIP-seq
☒	☐ Flow cytometry
☒	☐ MRI-based neuroimaging

Unique biological materials

Policy information about availability of materials

Obtaining unique materials	Seeds from all genotypes described herein are available through the Juenger laboratory.
